# An Update on Antimicrobial Peptides (AMPs) and Their Delivery Strategies for Wound Infections

**DOI:** 10.3390/pharmaceutics12090840

**Published:** 2020-09-02

**Authors:** Viorica Patrulea, Gerrit Borchard, Olivier Jordan

**Affiliations:** 1 Institute of Pharmaceutical Sciences of Western Switzerland, University of Geneva, 1 Rue Michel Servet, 1211 Geneva, Switzerland; gerrit.borchard@unige.ch; 2 Section of Pharmaceutical Sciences, University of Geneva, 1 Rue Michel Servet, 1211 Geneva, Switzerland

**Keywords:** bacterial infection, non-healing wounds, antimicrobial resistance, multidrug resistance, antimicrobial peptides (AMPs), AMP conjugates, AMP carriers and delivery systems

## Abstract

Bacterial infections occur when wound healing fails to reach the final stage of healing, which is usually hindered by the presence of different pathogens. Different topical antimicrobial agents are used to inhibit bacterial growth due to antibiotic failure in reaching the infected site, which is accompanied very often by increased drug resistance and other side effects. In this review, we focus on antimicrobial peptides (AMPs), especially those with a high potential of efficacy against multidrug-resistant and biofilm-forming bacteria and fungi present in wound infections. Currently, different AMPs undergo preclinical and clinical phase to combat infection-related diseases. AMP dendrimers (AMPDs) have been mentioned as potent microbial agents. Various AMP delivery strategies that are used to combat infection and modulate the healing rate—such as polymers, scaffolds, films and wound dressings, and organic and inorganic nanoparticles—have been discussed as well. New technologies such as Clustered Regularly Interspaced Short Palindromic Repeat (CRISPR)-associated protein (CRISPR-Cas) are taken into consideration as potential future tools for AMP delivery in skin therapy.

## 1. Introduction

Skin wound healing is a complex and highly orchestrated process that consists of four overlapping stages, namely inflammation, proliferation, migration, and the maturation of the new tissue [1,2]. If any of these phases is disturbed, healing may lead to either chronic non-healing wounds, such as venous, diabetic, and pressure ulcers or pathological scaring and excessive fibrosis. Major factors leading to chronic wounds are ischemia and microbial colonization [3,4]. These types of wound infections may serve as a bacterial reservoir and cause high morbidity and costs [5]. Nowadays, microbial infections due to multidrug resistance (MDR) are an important threat, which needs to be seriously considered. MDR bacteria hinder wound healing, as most of the wounds would develop infections at some point. The most opportunistic and MDR pathogens, which have the ability to colonize the wound, are included in the ESKAPE bacterial collection (*Enterococcus faecium*, *Staphylococcus aureus*, *Klebsiella pneumonia*, *Acinetobacter baumannii*, *Pseudomonas aeruginosa* and *Enterobacter* species) [6]. *S. aureus* and *P. aeruginosa* have additionally the ability to develop a biofilm on the wound and medical devices, which worsens the situation and makes biofilm bacteria extremely difficult or even impossible to treat. This is attributed to the fact that bacteria within biofilms are 100 to 1000-fold more tolerant to antimicrobial agents, thus delaying the healing of infected wounds [7]. Additionally, bacteria within biofilms are enclosed in a 3D network of a self-produced matrix of extracellular polymeric substances (EPS), such as exopolysaccharides, proteins, extracellular DNA, and teichoic and lipoteichoic acids, which amplifies the resistance to most of the existing antimicrobial agents [8]. Infected wounds are an alarming cause of death, especially in immunocompromised and diabetic patients, which poses a significant clinical and economic burden for the patients and the healthcare system.

In these contexts, antimicrobial peptides (AMPs) are used as promising alternatives to counter bacterial infections and control microbial spreading. As antimicrobial resistance (AMR) is currently becoming a worldwide threat, the main focuses of research are: (i) to identify existing or new AMPs and to characterize their efficacy against prevalent microorganisms, including their mechanism of action and toxicity toward mammalian cells and/or blood cells; (ii) to assess the role of AMPs in modulating the pro-inflammatory cytokines and in adaptive immune mechanisms; and (iii) to identify new approaches for AMPs delivery [9].

The main goal of this review is to identify and discuss potent AMPs that are able to eradicate MDR bacteria in wound infections and AMPs acting as wound-healing promotors. AMP dendrimers (AMPDs), similar to AMPs, are very potent against the ESKAPE collection and biofilm-related bacteria; these are discussed as well. We also review strategies to deliver AMPs topically—their covalent coupling, self-assembly, coupling to antibiotics, embedding into 3D scaffold or dressing, the use of nanotechnological platforms (i.e., organic or inorganic nanoparticles) and smart nanomaterials—as future promising technologies to deliver AMPs efficiently to the site of infection, and CRISPR-Cas are also discussed.

## 2. Antimicrobial Peptides (AMPs) and AMP Dendrimers (AMPDs)

AMPs are small molecules having a broad activity to treat microbial infections, especially those causing AMR [10]. They have several advantages over current antibiotic treatment, which we will further detailed and which makes them good candidates especially for the treatment of topical and systemic infections. The selection of AMPs for the treatment of different infectious diseases may start with an in vitro/in silico screening to determine their biological activity against different microbial strains. An in silico approach refers to the bioinformatics tools that use different algorithms to classify, predict, and design new AMPs [11,12]. The rational of in silico design relies on its potential to drastically reduce the costs for AMPs production and the time to evaluate their biological activity and potential toxicity by predicting in advance the bioactivity of the AMPs [6]. This methodology was shown to have a 94% accuracy in terms of biological activity and could predict which AMPs would be active in vitro [13]. Kumar Meher et al. developed a support vector machine (SVM)-based computational approach for the prediction of AMPs with an improved accuracy [14]. The most advanced computational tools to predict the biological activity of the AMPs with the best accuracy have been described in depth by different authors in the literature [12,15,16,17]. These “tailored” AMPs could help overcome microbial resistance and improve pathogen killing.

### 2.1. AMPs: Classification, Mechanism of Action

Antimicrobial peptides (AMPs), also called host defense peptides (HDPs), are found in bacteria, fungi, plants, and animals. They typically consist of 10–50 amino acid residues (very rarely up to 100 amino acids) and generally possess cationic (net charge ranging from −4 to +20) and amphipathic structures. They were identified for the first time in 1922, almost a century ago, by Alexander Fleming, who discovered lysozyme [18], an antibacterial enzyme found in saliva, tears, and human urine. Soon after, in 1928, he discovered penicillin, the first antibiotic in human history, which was extracted from the culture of green mold, *Penicillium notatum* [19]. Since then, more than 3171 AMPs have been reported according to the Antimicrobial Peptide Database (APD) [20]. According to the DRAMP (data repository of antimicrobial peptides, an open access and manually annotated database), there are currently 20,227 general entries related to AMPs, of which 5412 are general AMPs (including natural and synthetic AMPs), 14,739 entries are patented AMPs, and 76 are AMPs that are under development as drugs (preclinical or clinical phase), most of them having antimicrobial activity [21]. The main focus of the DRAMP database is to survey the hemolytic activity of AMPs, including detailed test protocols essential to evaluate the efficacy/toxicity balance of the AMPs [22]. 

**Figure 1:**
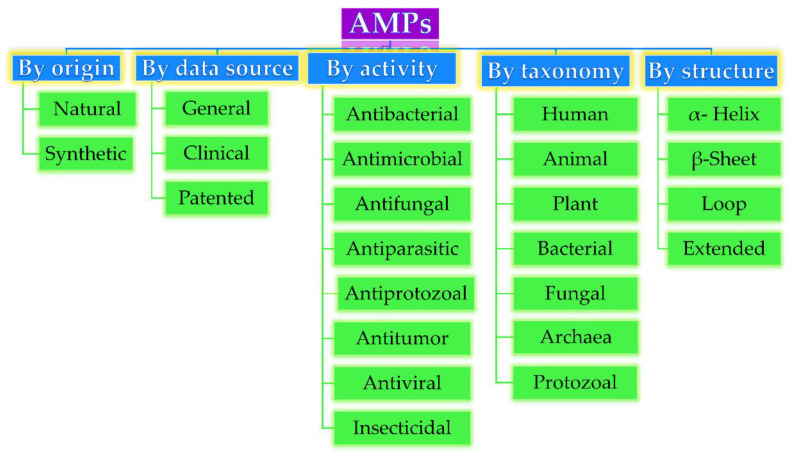
Antimicrobial peptides (AMP) classification (partially inspired by the DRAMP database [21]). DRAMP: data repository of antimicrobial peptides, an open access and manually annotated database.

Based on their origin, data source, activity against microorganisms, taxonomy, conformational structure, and amino acid composition, AMPs can be divided into several categories, as depicted in Figure 1. Very often, AMPs are classified based on their secondary conformation into four different families: α-helix, β-sheet, loop, and extended family. Most of the AMPs fall into the α-helix (e.g., cryptdin-4, human α-defensins (HD-5 and 6), magainin 1 and 2, melittin, moricin) or β-sheet families (e.g., human β-defensins (hBD-1-6), lactoferricin B, protegrin-1, tachyplesin I) [23]. Very few AMPs show a loop structure (e.g., thanatin), and extended structures with neither α-helical nor β-sheet conformation (e.g., Indolicidin, Indolicidin analogue (CP10A), tritrpticin) [6]. 

The biological activity of AMPs against both Gram-positive (Gram (+)) and Gram-negative (Gram (−)) bacteria, viruses, and fungi has drawn special attention, particularly their ability to kill MDR bacteria [24]. AMPs act mostly by disrupting the integrity of the cell membrane, which is accompanied by the leakage of vesicles and other cellular components within a very short time. Typically, their interaction is based on the electrostatic attraction between the negatively charged bacterial wall and positively charged AMPs [25]. Subsequently, once the interaction is established, AMPs access the phospholipid bilayer membrane and start to aggregate, leading to the formation of different complex structures through at least four distinct mechanisms, such as the formation of “aggregate”, “toroidal pore”, “barrel-state”, or “carpet” models, or via membrane permeabilization that would lead finally to cell lysis. These mechanisms are described in detail by Rios et al. [10] and Thapa et al. [3]. In addition to these bilayer membrane mechanisms, AMPs can diffuse via cytoplasmic membrane and accumulate intracellularly, subsequently blocking DNA replication and disrupting RNA and protein synthesis, which in turn leads to cell wall lysis. Buforin II, pleurocidin, and dermaseptin are examples of those [26]. A recent study showed that some AMPs such as cathelicidins [27] and papiliocin [28] can act via the production of reactive oxygen species (ROS) and mitochondrial dysfunction, thus leading to cell apoptosis. 

#### 2.1.1. Wound-Healing Promoting AMPs

AMPs not only exhibit a broad spectrum of antibacterial activity, but many of them display antibiofilm, anti-methicillin-resistant *Staphylococcus aureus* (MRSA), anti-tuberculosis, anti-sepsis, anti-toxin, antiviral, anti-HIV, antifungal, antiparasitic, anticancer, anti-diabetic, wound healing and anti-inflammatory activities as well. Some examples of AMPs split according to their activity are listed in Table 1. 

Many AMPs, beside their antimicrobial activity, additionally promote wound healing and stimulate angiogenesis, which are key factors in the process of tissue regeneration. Several AMPs that have shown promising results after topical application, both in vitro and in vivo, are mentioned in Table 1. For instance, LL-37 (phase II in clinical trials) was shown to promote angiogenesis, migration, and the proliferation of dermal cells, which is a crucial factor in wound restoration [29] and used for treatment of diabetic foot ulcer (DFU). IDR-1018, an innate defense regulator peptide and less toxic than LL-37, was shown to significantly accelerate wound healing in non-infected and non-diabetic murine and *S. aureus*-infected porcine models [30]. Human β-defensin (hBD-2) induces keratinocyte migration, while hBD-3 has been additionally reported to accelerate wound closure with a 10-fold bacterial reduction in a porcine model of *S. aureus*-infected diabetic wounds [31]. Furthermore, hBD-3 and LL-37 were shown to stimulate not only dermal cells, but also corneal epithelial cells with an enhanced ocular surface healing [32]. LL-37 induced the migration, proliferation, and wound closure of airway epithelial cells [33]. Pexiganan, an analogue of magainin 2 and the first AMP evaluated for human skin infections, was shown to promote dermal cell migration and antibacterial activity against Gram (+) and Gram (−) cells (minimal inhibitory concentration (MIC) ≤16 µg/mL) in diabetic foot ulcer models early in clinical trials. Consequently, Locilex^®^, a 0.8% pexiganan cream, was patented and developed for DFU treatment. Locilex^®^ failed later during phase III of clinical trials due to a lack of efficacy compared to placebo or “vehicle” cream without pexiganan [34]. 

The lack of stability of AMPs is an important issue, and most of the AMPs could self-assemble, losing their activity even in a saline solution. Furthermore, they may be sensitive to enzymatic degradation [35]. For instance, Nishikawa et al. developed a helical AMP, AG-30, which has angiogenic properties in a mouse ischemic limb model. AG-30 exhibited high antimicrobial activity against *P. aeruginosa* (MIC: 5 µg/mL), *E. coli* (MIC: 40 µg/mL), and *S. aureus* (MIC: 20 µg/mL) via a membrane disruption mechanism [36]; however, it was lacking stability in saline. An improved version of AG-30, in terms of stability, is the cationic AG-30/5C, which was developed by replacing five residues of AG-30 with five cationic amino acids. It is noteworthy that AG-30/5C was shown to be stable in a saline solution for at least 12 months at 5 °C. In vitro, AG-30/5C showed a strong antimicrobial activity against *P. aeruginosa* (MIC: 5 µg/mL), *S. aureus* (MIC: 50 µg/mL), MRSA (50 µg/mL), and *Candida* (MIC: 15.5 µg/mL). In vivo, AG-30/5C accelerated re-epithelialization and angiogenesis in MRSA-infected diabetic mouse and porcine models [37]. AG-30/5C significantly improved the blood flow around the wound compared to the LL-37 control group. Another cationic peptide, AH90, isolated from the skin of frogs, promoted wound healing by stimulating transforming growth factor (TGF-β1) in a full-thickness mouse model. The native AH90 peptide was shown to promote faster wound shrinking in mice compared to a positive control, EGF (epidermal growth factor) and the scrambled version of the peptide, called sAH90 [38]. 

**Figure 2:**
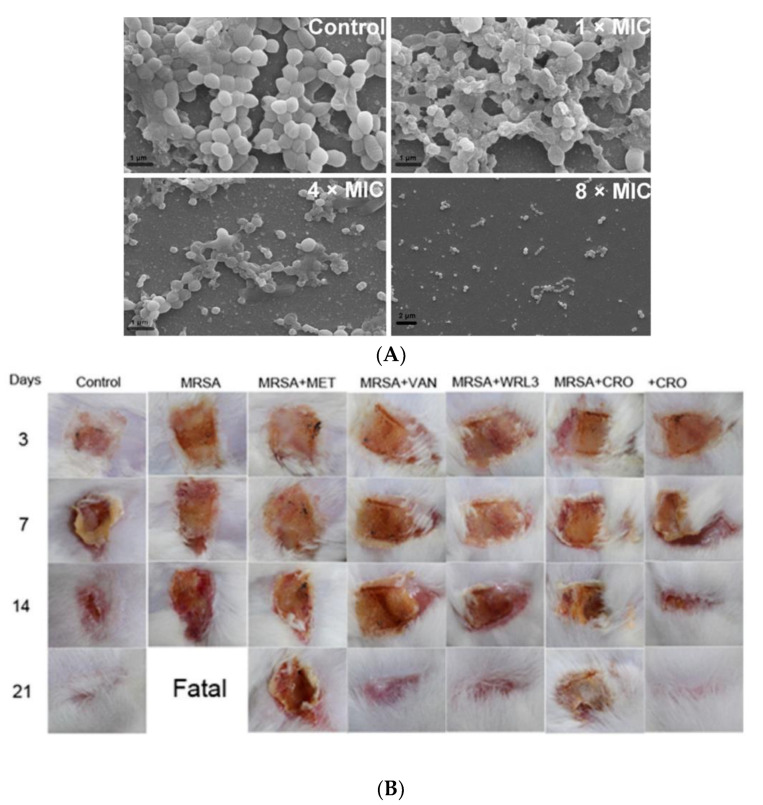
Antibacterial activity of WRL3 and antibiotics. (**A**) SEM images of the in vitro methicillin-resistant *S. aureus* (MRSA)-biofilm inhibition at indicated VWR3 concentrations compared to the control cells; (**B**) Photos of wound regions of approximately 1 cm^2^ on the back of MRSA-infected mice and treated with MET (methicillin), VAN (vancomycin), WRL3, CRO (ceftriaxone), or WRL3 and CRO at 3, 7, 14, and 21 days post-MRSA infection. “Fatal” indicates no mice survival. From [41] with permission from the American Chemical Society© Copyright 2020.

Tylotoin promotes both wound healing via the induction of TGF-β1 and IL-6 secretion, which are responsible factors for wound healing, and angiogenesis by inducing endothelial cell tube formation. Enhanced cell motility and the proliferation of both keratinocytes and fibroblast in vitro and the formation of granulation tissue at the wounded site in a murine model of full-thickness was shown as well [39]. A small peptide, tiger17 (11-mer), promoted wound healing in vitro and in vivo. It enhanced the migration and proliferation of keratinocytes and significantly accelerated wound closure in a murine full-thickness wound model. Tiger17 induced macrophage recruitment to the inflammation site and via the migration and proliferation of keratinocytes, it induced re-epithelialization and granulation tissue formation. Furthermore, tiger17 stimulated the release of TGF-β1 and IL-6, which are responsible for wound regeneration [40]. A relatively small peptide, WRL3 (18-mer), exhibited high cell selectivity by its ability to specifically eliminate MRSA cells (MIC: 2 μg/mL) grown in a co-culture model via the membrane lysis mechanism and efficiently eradicating biofilms of MRSA. In vivo, WRL3 successfully removed bacteria, stimulated pro-inflammatory cytokine secretion, recruited macrophages, and stimulated angiogenesis and wound closure in an MRSA-infected burn mouse model (Figure 2). WRL3 showed to be more efficient than vancomycin in reducing MRSA infections and to reduce the healing time in burn wounds [41]. Therefore, WRL3 might be used not only for wound healing promotion, but also for treating MRSA-related infections in skin burn wounds. 

Another potent AMP, epinecidin-1 (Epi-1), induced keratinocytes proliferation and migration in vitro. In vivo, Epi-1 induced complete healing in an MRSA-infected heat-burned pig skin over a period of 25 days. The results showed that Epi-1 enhanced vascularization, increased epithelial activities, and stimulated collagen synthesis around the wound region [42]. 

Therefore, AMPs are considered to have a high potential in eradicating microorganisms but as well in promoting wound healing via stimulating re-epithelialization, angiogenesis, collagen, and granulation tissue formation. 

The main hurdle of AMPs is their short elimination half-life due to their fast degradation in the presence of proteases once in the blood stream [43]. Therefore, most of the currently used AMPs are directed for topical delivery, especially to wounds. Routinely used AMPs colistin, dalbavancin, daptomycin, oritavancin, and telavancin have a half-life of 5 h, 8 h, 8 h, 14 days, and 195.4 h, respectively, while the half-life of gramicidin has not been determined yet. On average, the median half-life of FDA-approved peptides is 9 h [44]. As the main focus of this review is on the topical application of AMPs, we considered AMPs, which are in their preclinical and clinical trials development stage, as well those routinely used FDA-approved AMPs and potent AMPs for topical wound infections, which possess antibacterial or in specific cases antifungal activity (Table 2). 

Currently, at least 10 AMPs failed in their clinical phases [3]. As examples, pexiganan (Locilex^®^), which was used for treatment of infections in diabetic foot ulcers, failed during clinical phase III. Iseganan (IB-367, protegrin-I analogue), used for preventing polymicrobial oral infections such as stomatitis, failed in phase III as well. Omiganan (indolicidin derived; MBI-226) failed during phase III due to catheter-related infections [10]. XMP-629 was used to treat impetigo and acne rosacea, and Murepavadin (POL7080) [72], a protegrin analogue used to treat ventilator-associated bacterial pneumonia, was halted recently in phase III. These failures were attributed to the lack of significant efficacy compared to other antibacterial drugs or to multiple side effects [23].

#### 2.1.2. The Challenge of Resistance Development 

All types of pathogens tend to develop their own defense mechanism [62], adapting to their specific environment. Therefore, it is unrealistic to expect no development of resistance to AMPs. Generally, microbes create resistance via different modalities, such as modification of their structure or electrical charge [89], modification in the cell wall and metabolism, LPS myristylation, acylation of lipid A, etc. [90]. Additionally, microbes adapt to survive at high ionic concentration, which is a condition known to hinder AMP potency [62].

However, it should be noted that bacteria will develop resistance to AMPs at a much slower rate than to antibiotics [91]. This is attributed to the relatively low specificity of AMP mechanisms of action, as well as to the variety of these mechanisms implied in bacterial killing. In general, AMPs are quite resistant to the development of bacterial resistance [69].

#### 2.1.3. AMP’s Activity against Biofilm Formation

Although AMPs show high activity against a broad spectrum of bacteria, very little is known about the resistance mechanisms developed by biofilms. Biofilm formation by bacteria is another critical issue for infectious diseases. Most pathogens form microcolonies by adhering to each other and then produce a biofilm as a protective environment. Biofilm formation is initiated by (i) planktonic cell attachment to a surface; (ii) colony formation; (iii) biofilm formation, when bacterial colonies are anchored tightly within a matrix of extracellular polymeric substances (EPS), which includes extracellular DNA, proteins, lipids, and polysaccharides; and (iv) cell dispersion [92]. Further, the developed biofilm dispersion may lead to the spread of infection and new colonization sites and may create increased antimicrobial resistance. 

Severe infections are associated with biomedical devices and implants due to their high susceptibility to bacterial colonization [93]. Very often, implant surfaces become a reservoir of bacteria that can spread very fast into the whole body, which leads to persistent chronic infections. The only way to eradicate bacteria in this situation is implant removal, which implies surgery and close follow-up of the patients for their complete recovery. In this context, it is worth noticing that AMPs, such as AG-30 [36], AG-30/5C [37], WRL3 [41], melimine and Mel4 [69], 73c, and D-73 [94] are very potent against biofilm formation. 

### 2.2. Antimicrobial Dendrimer Peptides

**Figure 3:**
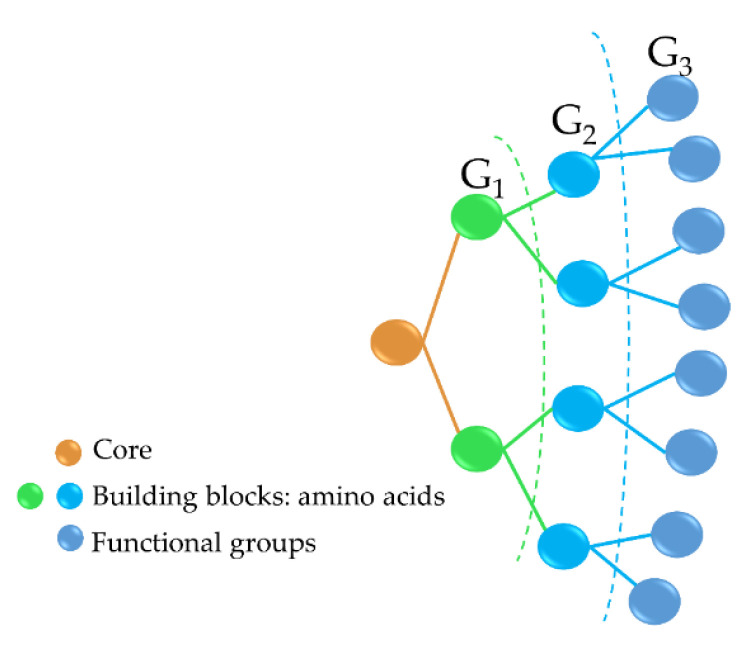
Third-generation AMPD structure, including the dendrimer core (peptidic or non-peptidic), building blocks (amino acids), and functional groups (peptidic or non-peptidic) for coupling other bioactive molecules. G_1_, G_2_, and G_3_ indicate the different generation levels.

Antimicrobial peptide dendrimers (AMPDs) are highly branched 3D structures with a central core and high density of flexible surface groups for potential molecule attachment [95]. They can be obtained by solid-phase peptide synthesis (SPPS) and purified by HPLC to ensure high reproducibility [96]. Compared to polymeric dendrimers, they are built on covalently coupled amino acid residues [92]. AMPDs can be built up to several generations based on the layers of branching units from G_0_ to G*_x_*, where *x* represents the number of branching cycles (Figure 3). They are a very attractive class of molecules as they exhibit high antimicrobial activity against bacteria, fungi, and viruses [97]. Their high antimicrobial activity is mainly due to their positive charges of the amino acid residues that interact with the negatively charged bacterial cell envelope and lead to bacterial death [92,98]. Moreover, AMPDs were shown to be more resistant to proteolysis and less toxic to both mammalian and erythrocyte cells than AMPs.

In addition to their intrinsic activity and due to the fact that AMPDs have different functional groups, it makes them very attractive delivery systems as drug carriers. Increasing the number of ramification leads to an increase in functional groups, thus a higher opportunity to couple different bioactive molecules [95].

Bacterial killing by AMPDs is governed by functional groups of the amino acid residues and their ability to penetrate the cell membrane [92]. A recent example of effective third generation (G_3_) AMPD, G3KL (incorporating repetitive lysine (K) and leucine (L) units), showed high activity against 4 out of 6 in the ESKAPE collection in 32 different strains of *A. baumannii* (MIC: 16 µg/mL), 35 strains of *P. aeruginosa* (MIC: 8–32 µg/mL) [99], *E. coli* (8 µg/mL), and *K. pneumoniae* (16–64 µg/mL) [96]. The mechanism of action is based on membrane disruption followed by vesicle leakage as shown by transmission electron microscopy (TEM) analysis. G3KL AMPD showed as well to inhibit *P. aeruginosa* biofilm formation [100]. Fluorescently labeled G3KL entirely diffused into *P. aeruginosa* within 15 min as shown in a time-lapse assay, suggesting AMPD diffusion or translocation into the cytoplasm through damaging both the outer and inner bacterial membrane, as was confirmed by TEM [101].

**Figure 4:**
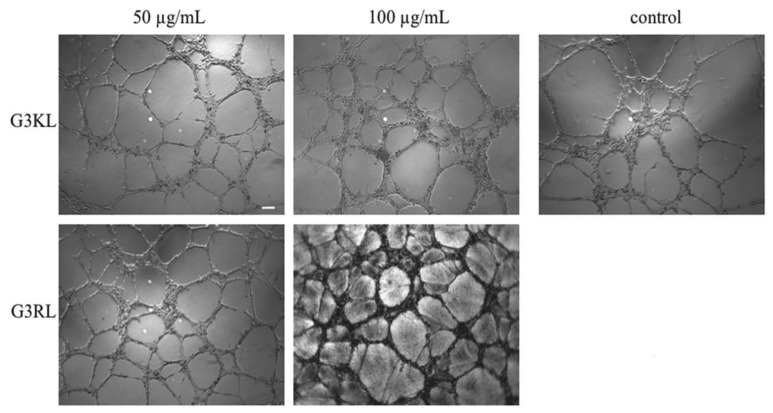
Images of the endothelial tubular networks in Matrigel after 5 h treatment with G3KL (incorporating repetitive lysine (K) and leucine (L) units), and G3RL (consisting of arginine (R) and leucine (L) repeats) at indicated concentrations versus PBS (Phosphate-Buffered saline) control. From [5] with the permission by Springer Nature©, Copyright 2016.

G3KL and another G_3_ (G3RL, consisting of arginine (R) and leucine (L) repeats) showed high antibacterial activity against *P. aeruginosa* and neither toxicity nor gene alteration toward progenitor fibroblasts at an elevated concentration of 100 µg/mL. Furthermore, G3KL better promoted angiogenesis than G3RL in a human umbilical vein endothelial cells (HUVEC) and chorioallantoic membrane (CAM) model (Figure 4) [5], which is an important aspect for accelerated wound healing. 

A second generation (G_2_) AMPD, TNS18, showed the same activity as G3KL against *P. aeruginosa* (MIC: 8–16 µg/mL), a much higher activity against *S. aureus* (MIC: 8–16 µg/mL), but less potency against *K. pneumoniae* than G3KL [96]. Combination of the peripheral branches of G_2_ (TNS18) with the core of a G3 (called T7) resulted in a chimeric AMPD named DC5. DC5 displayed significant activity against *K. pneumoniae* (MIC: 16–32 µg/mL), MRSA (MIC: 32 µg/mL), *E coli* (MIC: 8 µg/mL), *A. baumannii* (MIC: 16 µg/mL), and *P. aeruginosa* (MIC: 4–8 µg/mL) strains [96]. In vivo on *larvae*, *D*-enantiomers of *d*G3KL and *d*TNS18 showed a high ability to kill *P. aeruginosa* biofilm (killing efficiency 90.2–100%) and similar to tobramycin [102].

Another family of AMPDs based on R4 tetrapeptide (RLYR repetitive units) and R8 octapeptide (RLYR-KVYG repeats) was tested against 10 different microbial strains (*E. coli*, *P. aeruginosa*, *P. vulgaris*, *K. oxytoca*, *S. aureus*, *M. luteus*, *E. faecalis*, *C. albicans*, *C. kefyr*, and *C. tropicalis*) with lower MICs than 1 µM. Noteworthy, they proved as well to be more resistant to proteolysis and more non-hemolytic than the corresponding linearly repeating peptides [97]. A SB056 lipodimeric AMPD exhibited high microbicidal activity against both Gram (+) and (−) bacteria with an MIC as low as 2–32 µg/mL against *A. baumannii*, *E. cloacae*, *E. coli*, *K. pneumoniae*, and *P. aeruginosa*, which is comparable activity to polymyxin B. Importantly, SB056 strongly inhibited *E. coli*, *S. aureus*, and *S. epidermis* biofilm formation [103].

These AMPDs with preferred selectivity have proven to be highly active against the whole range of ESKAPE bacterial pathogens and others, including fungi as well. Additionally, SB105-A10 AMPD exhibits high antiviral activity against human papillomaviruses, human respiratory syncytial virus (RSV), and human immunodeficiency virus type 1 (HIV-1), as described elsewhere [95]. Therefore, further studies are warranted to bring good news in the fight against AMR in infection-related diseases. So far, there are no further studies with regard to the coupling of AMPDs with AMPs or other chemistry involved to deliver AMPDs to the wound site.

## 3. Peptide Conjugates 

Needless to mention, most of the recent studies report on new strategies to combat MDR and biofilm formation. It is clear now that the antimicrobial activity could be considerably enhanced by several strategies, such as the chemical addition of different molecules via covalent grafting, incorporation into different nanosystems, self-assembly, coupling with antibiotics, and incorporation into polymer scaffolds, while maintaining or improving AMP’s antibacterial activity with prolonged blood circulation and reduced cytotoxicity. Other approaches for “tailoring” AMPs with enhanced stability, such as modification of the C- and N-terminus via acetylation and amidation, cyclisation, lactamization, lactonization, macromolecular conjugation, and PEGylation have been discussed elsewhere in detail [62,104,105]. 

Another potential strategy to improve the efficacy of current antibacterial agents is to use antibiotic resistance breakers (ARBs). ARBs are a type of antibiotic adjuvants that are capable of re-sensitizing resistant bacteria to antibiotics. The three main classes of ARBs include β-lactam inhibitors, membrane permeabilizers, and efflux pump inhibitors (EPIs) [106,107]. Among ARB–antibiotic combinations, the co-administration of β-lactamase inhibitors (e.g., clavulanic acid) with β-lactam antibiotics (e.g., amoxicillin) has met clinical success and been extensively described in the literature [106,107,108].

### 3.1. Covalent Coupling to Polymers

Polymers are composed of several repeating units of monomers. Many natural polymers, such as proteins (i.e., collagen, elastin, keratin, and silk fibroin) and polysaccharides (i.e., alginate, chitosan, hyaluronic acid, and cellulose), or synthetic polymers (i.e., poly(lactic acid) (PLA), poly(vinyl alcohol) (PVAL), poly(caprolactone) (PCL), poly(lactic-*co*-glycolic acid) (PLGA), and poly(ethylene glycol (PEG) have been widely used for medical applications [109]. Natural biopolymers (e.g., chitosan, collagen, and hyaluronic acid (HA)) have been shown to promote wound healing, for instance by stimulating anti-inflammatory responses in chronic wounds [3]. A loss of antimicrobial activity or low availability at the implant site when antimicrobial agents are covalently coupled to different surfaces is a known problem related to antimicrobial coatings [110]. To overcome these issues, AMPs are promising agents with high bioactivity and biocompatibility, which showed relatively low bacterial resistance.

Among biopolymers, chitosan is widely used thanks to its amino and hydroxyl functional groups [111,112], which offer a variety of possibilities for covalent or non-covalent coupling. Chitosan is a biocompatible, biodegradable polymer with antimicrobial activity and the ability to promote wound healing, dermal cell proliferation, and migration [111,112]. Several chitosan–AMP conjugates have been designed, such as Dhvar-5 AMP immobilized to chitosan via azide-alkyne or “click” reaction, which showed promise as antibacterial coatings to prevent biomaterial-related infections. An ultrathin layer was obtained by spin coating when dissolving AMP–chitosan in acetic acid. Higher antibacterial (MIC: 4, 2, 16, and 4 µg/mL for *S. aureus*, *S. epidermidis*, *E. coli*, and *P. aeruginosa*, respectively) and antibiofilm activities were found when coupling Dhvar-5 peptide via C-terminus than when coupling through the N-terminus, underlining the importance of the conformation of the exposed peptide. Moreover, the AMP–chitosan conjugate did not show any cytotoxicity to fibroblasts [113]. Similarly, hLF1-11 AMP was covalently immobilized by C-terminal onto chitosan thin films; the presence of hLF1-11 inhibited the growth of MRSA in comparison to the control [64]. In another study, cysteine-modified MSI-78 AMP was covalently immobilized to chitosan coatings through a PEG linker. MSI-78–chitosan films showed high antimicrobial efficacy against *S. epidermidis* in both PBS and PBS supplemented with 1% human plasma, maintaining significantly low bacterial adhesion, while killing 76% of adherent bacteria [114]. This type of coating could be successfully used for medical applications for implants or wound dressings.

**Figure 5:**
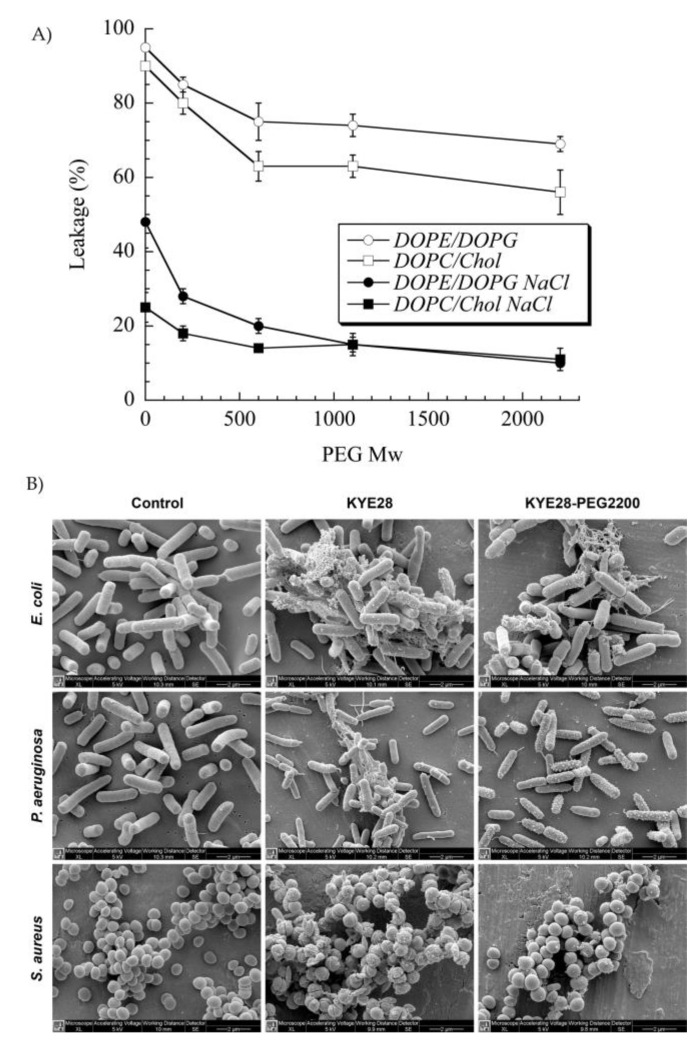
(**A**) Effect of poly(ethylene glycol (PEG) length on KYE28–PEG leakage induction of DOPE/DOPG (75/25 mol/mol) and DOPC/cholesterol (60/40 mol/mol) liposomes at a peptide concentration of 1 μM; (**B**) Peptide-mediated permeabilization of *E. coli*, *P. aeruginosa*, and *S. aureus* with the indicated peptides at 30 μM for 2 h, and analyzed by SEM. DOPE: 1,2-dioleoyl-*sn*-glycero-3-phosphoethanolamine, DOPC: 1,2-dioleoyl-*sn*-glycero-3-phosphocholine, and DOPG: 1,2-dioleoyl-*sn*-glycero-3-phosphoglycerol, monosodium salt. Reprinted from [118] with the permission of American Chemical Society©, Copyright 2014.

PEG has been extensively used to functionalize peptides, drugs, and nanoparticles. This process of PEGylation offers different advantages, such as increased resistance to degradation, increased blood circulation time, reduced aggregation, and reduced toxicity [115]. Sometimes, peptide PEGylation may lead to reduced proteolysis and toxicity at a cost of lower antibacterial effect. As such, Imura et al. PEGylated tachyplesin I and found lower antimicrobial activity than the non-PEGylated peptide against *E. coli* and significantly lower toxicity against CHO-K1 cells [116]. In another study, the same authors loaded unilamellar vesicles with PEGylated magainin 2 and found that PEG-magainin 2 had weaker vesicle leakage, lower bactericidal activity, and significantly lower toxicity to CHO-K1 cells [117]. To investigate this decrease in antibacterial activity, Singh et al. PEGylated the KYE28 AMP using PEG with different lengths and conjugation sites. They found that the antibacterial activity decreased against *E. coli* and *P. aeruginosa* and dramatically decreased toward *S. aureus* with the increase of PEG length, irrespective of the conjugation site. Cytotoxicity and hemolysis were strongly reduced as well, while selectivity was improved (Figure 5) [118]. Thus, PEGylation is an attractive technique to optimize the antibacterial efficacy of the peptide versus their toxicity. In another study, peptide 73, a derivative of aurein 2.2Δ3, coupled to PEG exhibited a 2- to 8-fold enhancement of antibacterial activity against *S. aureus* biofilms [94].

In general, the bacterial colonization of medical devices favors biofilm development, which in turn will lead to device failure due to peri-implantitis [119]. For instance, *P. aeruginosa* colonization of catheters and other medical devices accounts for 10–20% of all related infections [69]. Therefore, coating medical devices with a layer of antibacterial membrane or covalently attaching different AMPs to surfaces are advantageous strategies to combat biofilm formation and reduce the rate of infection. In this view, two cationic AMPs, melimine and Mel4 (melimine derivative), which are in phase II/III clinical trials, were covalently attached to a model glass surface [69]. Surfaces coated with both peptides lead to cytoplasmic bacteria leakage within 15 min. Moreover, the antimicrobial activity of melimine was preserved upon covalent coupling to polymers or titanium, and melimine-coated lenses did not show signs of conjunctival redness, although staining of human cornea was observed in some cases [120]. On the contrary, Mel4-coated lenses showed no signs of corneal staining or redness or ocular irritation [121]. Moreover, covalently coupled melimine and Mel4 to the glass surface was shown to successfully eradicate *P. aeruginosa* within 15 min [69]. Overall, AMP–polymer conjugates can confer effective antibacterial properties to the surfaces of medical devices at the preclinical stage.

### 3.2. Self-Assembled AMPs

Peptides can self-assemble into different nanostructures, such as micelles, nanotubes, vesicles, and fibrils. Their self-assembly mechanism is driven by the spontaneous organization of peptides based on their electrostatic or hydrophobic interactions, hydrogen bonding, or π–π stacking [122]. An important focus of self-assembled peptides research relates to the formation of amyloids, which is a key process for the onset of neurodegenerative diseases, especially with regard to the link between microbial infection and Alzheimer’s, Parkinson’s, Creutzfeldt–Jakob, or related diseases [123,124,125,126]. For example, Alzheimer’s β-amyloid diphenylalanine (FF) dipeptide was found to drive the self-assembly of FF into stiff nanotubes in Alzheimer’s disease [127]. A β-amyloid KLVFF peptide that has the same mechanism as FF peptides could self-assemble into nanofibrils and then into a nanofibrillar gel in a concentrated phosphate-buffered saline solution [128]. Additionally, amyloid peptides such as islet amyloid polypeptide (IAPP) exhibited antimicrobial activity, and sometimes, their activity was higher than that of LL-37 [129]. Therefore, AMPs could suggest the potential connection between amyloid and AMPs in combating amyloid-related neurodegenerative diseases. AMP may be of therapeutic interest in the treatment of neurodegenerative diseases, since persistent microbial infection is thought to be one of the triggering factors of these diseases.

Melittin, a very potent AMP extracted from bee venom but severely cytotoxic, has been co-assembled upon the addition of a molecular block containing synthetic multidomain peptides. Upon the self-assembly of melittin into nanofibers, membrane selectivity and cytocompatibility was significantly improved [130]. Yazici et al. combined a hydroxyapatite-binding peptide-1 (HABP1) with a tet127 AMP through a flexible linker as self-assembled antimicrobial agents against *E. coli* (MIC: 32 µg/mL) and less efficiently against *Streptococcus mutans* (MIC > 256 µg/mL). When using these self-assembled HABP1-tet127 to functionalize calcium–phosphate coated on nanotubular titanium surfaces, a better inhibition of *E. coli* (90%) than *S. mutans* (75%) was shown [131]. This type of self-assembled peptides could be used for functionalizing the surfaces of different medical implants for local therapy to prevent bacterial infection. Bacitracin A modified with poly(*D*,*L*-lactic-*co*-glycolic acid) (PLGA) and poly(ethylene glycol) (PEG) exhibited self-assembling properties with high antibacterial potency both in vitro and in vivo. All PEGylated self-assemblies showed significantly stronger antibacterial activity against Gram (+) and Gram (−) compared to the non-PEGylated system. The antibacterial activity for non-PEGylated self-assemblies was severely compromised due to low water solubility. In contrast, the PEGylation of bacitracin–PLGA self-assemblies significantly improved water solubility of the system and in turn, the antibacterial activity was improved against *E. coli* (MIC: 1 µM) and *S. aureus* (MIC: 0.5 µM) without significant toxicity in a mouse thigh infection model. Bacitracin in solution, used as control, was effective only against Gram (+) bacteria with MICs between 2 and 4 µM, while MICs against Gram (−) were higher than 128 µM. It was shown as well that PEGylation did not affect antibacterial activity; on the contrary, PEGylated bacitracin-PLGA exhibited high accumulation in inflammatory tissue and prolonged blood circulation [132]. This system could be efficiently used to design novel antibiotic nano-assemblies in the treatment of invasive infections.

Self-assembly is a promising strategy toward the design of smart materials that could incorporate antibiotics or other antimicrobial agents with stimuli-responsive properties. For example, pH-sensitive materials could release antibiotics at a pre-defined pH, ionic strength or temperature, enhancing activity in the targeted infected wounds. Moreover, due to their high biocompatibility, biodegradability, and membrane selectivity, AMP self-assemblies hold great potential toward eradicating MDR and biofilm-forming bacteria. Self-assembled peptides and factors influencing AMP self-assembly have been described by Malekkhaiat Häffner and Malmsten in detail [126].

### 3.3. Combination of AMPs with Antibiotics

AMPs associated with conventional antibiotics generally showed high synergism in terms of their antimicrobial activity [62]. This is attributed to the fact that the combined antibiotic/antimicrobial therapy causes bacterial cell membrane disruption, facilitating antibiotic penetration and accumulation in the cytoplasm, ultimately leading to cell death. For example, magainin 2 and cecropin A combined with rifampicin showed synergistic interactions by significantly inhibiting MDR *P. aeruginosa* development in vitro and in vivo in rats. The best in vivo results on mortality rates and bacteraemia were obtained when administering intraperitoneally the combination of rifampicin (10 mg/kg) with either magainin 2 (dose of 1 mg/kg) or cecropin A (1 mg/kg). Mice treated with rifampicin (10 mg/kg), magainin 2 (1 mg/kg), or cecropin A (1 mg/kg) alone showed a high mortality rate and high bacterial survival, respectively [133]. This finding suggested that the membrane-permeabilizing activity of the peptides allows rifampicin to gain access to its intracellular target. Stronger synergistic effects have been observed in vivo in a mouse model of sepsis than in vitro when combining tachyplesin III with imipenem [134]. Synergistic effects against MDR *P. aeruginosa* were seen when combining a peptide (18 mer) called “P5” with isepamicin. Isepamicin could enter bacterial cells and inhibit protein synthesis assisted by P5, which lysed the cell membrane [135]. Such synergy was not observed upon mixing P5 with cefpiramide. Thus, combinations of P5 with isepamicin may be used for the treatment of patients with cholithiasis, which is a condition affected by antibiotic-resistant bacteria.

Similarly, B2088 AMP in combination with different conventional antibiotics, such as chloramphenicol, tobramycin, gentamicin, and imipenem exhibit synergistic antibacterial effects against *P. aeruginosa* without cytotoxicity against mammalian cells [136]. For instance, the WRL3 peptide combined with ceftriaxone exhibited synergistic effects against MRSA-infected burn wounds in mice [41]. This AMP–antibiotic mixture could be efficiently used in clinical applications for burn wound infections, where MRSA and *P. aeruginosa* cause high rates of death.

Therefore, the combined administration of AMPs and antibiotics appears to be a promising strategy and is already applied in clinics for its synergistic antibacterial effects and ability to fight MDR bacteria causing infectious diseases. Additionally, AMPs/antibiotics synergies against difficult-to-eradicate biofilms have been reported [137,138,139]. 

## 4. Nanotechnological Platforms and Scaffolds for Peptide Delivery 

As mentioned before, stability, short half-life, and cytotoxicity are the key factors limiting most of the AMPs from further clinical application. During the last few decades, a plethora of studies have been performed to find different strategies or systems to deliver AMPs. Nanotechnology is the field of science that uses nanocarriers such as nanoparticles (NPs) as drug delivery systems. NPs are particles in the range of 0.1 to 100 nm. The conjugation of AMPs with NPs results in increased local concentration at the delivery site with an enhanced AMP bioactivity, which might be attributed to a synergistic effect [140]. Nanotechnology can add many advantages, such as the improvement of solubility, bioavailability, release, and higher penetration within biofilms of AMPs [141,142]. They can provide protection to AMPs against degradation in different environments, such as enzymatic degradation [35].

### 4.1. Polymeric Scaffolds and Wound Dressings

Polymer scaffolds are the most used formulations to address wound healing and recovery due to their ability to promote cell attachment and proliferation, extracellular matrix generation, and the restoration of vessels via creating physical bonds between the cells and 3D network of the scaffolds [143]. Moreover, scaffolds can retain a significant amount of water or biological fluids within their 3D network due to the interaction of water with polymer hydrophilic groups (hydroxyl, amine, amide, and carboxyl groups), or hydrophobic interactions with specific biological fluid components and/or osmotic driving force effects [144]. Polymer-based scaffolds can be applied at the wound site and be used as reservoirs for the delivery of AMPs via diffusion, swelling, or chemical degradation [3,145].

**Figure 6:**
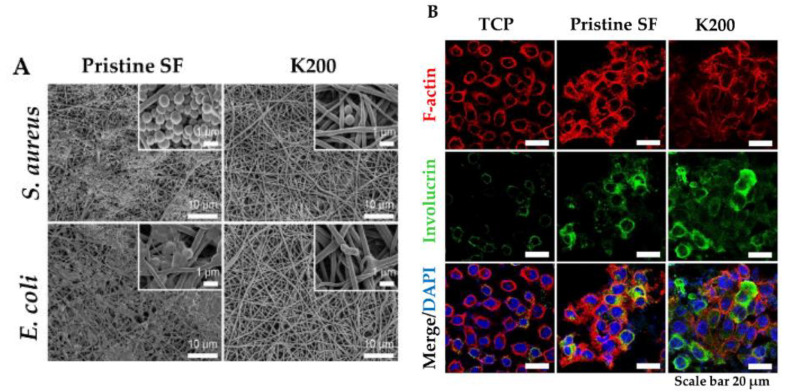
(**A**) FE-SEM images of *S. aureus* and *E. coli* cultured on Pristine SF and Cys-KR12 at 200 µg/mL (K200); (**B**) Confocal immunofluorescence images of keratinocytes cultured on TCP (tissue culture treated plates), pristine SF and K200 (red channel, F-actin; green, involucrin; blue, DAPI (4′,6-diamidino-2-phenylindole)). Reprinted from [45] with permission from Acta Materialia Inc.©, Copyright 2016.

In this view, a new strategy for topical application was developed by tethering human cathelicidin LL-37 on collagen scaffolds for the treatment of wound infections. LL-37-loaded scaffolds exhibited no toxicity toward fibroblast at a peptide concentration 24-fold higher than the cytotoxic threshold [146]. After 14 days, LL-37 loaded onto collagen domains (derived from collagenase or fibronectin) was retained on the scaffolds and showed no inhibition of antimicrobial activity against both Gram (+) and (−). This poor delivery may be due to too strong peptide–scaffold interactions. Similarly, Cassin et al. evaluated the incorporation of LL-37 into collagen and hyaluronic acid polyelectrolyte multilayers (PEMs) via physisorption and covalent immobilization [147]. LL-37-loaded thin films effectively prevented *E. coli* adhesion, but cells treated with covalently immobilized AMP presented morphological changes, suggesting cytotoxicity. In another study, Cys-KR12 AMP, a LL-37 derivative, was covalently immobilized on electrospun silk fibroin (SF) nanofiber membranes via EDC/NHS (1-Ethyl-3-(3-dimethylaminopropyl)carbodiimide/ N-hydroxysuccinimide) and thiol-maleimide “click chemistry”. Chemical immobilization of the AMP to SF membranes did not inhibit the biological activity of the peptide. On the contrary, the conjugate was highly active against four different strains, such as *S. aureus*, *S. epidermidis*, *E. coli*, and *P. aeruginosa*, with no biofilm formation (Figure 6) [45]. Cys-KR12-SF membranes promoted the proliferation of keratinocytes and fibroblasts, and they induced the differentiation of keratinocytes with a pronounced cell adhesion, which are important key steps in wound healing. Cys-KR12 (20 μg/mL) was used as positive control. These membranes are promising candidates for topical wound-healing applications.

Cellulose and its derivatives loaded with AMPs have been tested in diabetic foot ulcer (DFU) treatment. In a randomized phase III clinical trial [148], ACT1 AMP embedded in a hydroxyethyl–cellulose (HEC) hydrogel (Granexin) was topically applied for DFU treatment. The results showed that Granexin treatment was safe and effective for DFU treatment with a significant reduction of the ulcer area at week 12 (72% reduction versus control with 57%). All patients treated with ACT1-based hydrogels reached 100% ulcer re-epithelialization in a short time with no toxicity, no immunogenicity, or any side effects reported [149]. Such systems confirm their utility for the treatment of topical wound infections, especially DFU patients.

All these studies support the potential for AMP loaded into polymer scaffolds for the sustained and enhanced biological activity of the AMPs. Designing a polymer-based scaffold loaded with AMP that could control the AMP release via binding to the matrix, while promoting wound healing via the bioactivity of the scaffold itself, would be beneficial not only for wound healing, but also for preventing bacterial infection.

### 4.2. Organic Nanoparticles

#### 4.2.1. Polymeric Nanoparticles, Nanoemulsions, and Micelles

Polymer nanoparticles may be produced either in the form of gel-like particles or solid polymer particles. Nano- or microgel particles are prepared from swollen hydrophilic polymers; they are generally obtained by ionotropic gelation, a one-step process in which a counter-ion is added to a polyelectrolytic polymer, thus producing micro- or nanoparticles though ionic interactions. In contrast, solid polymer particles are based on organo-soluble, poorly hydrophilic polymers such as PLGA or PLA, and they may be produced by methods such as oil-in-water or water-in-oil-in-water emulsion evaporation. 

Carboxymethyl chitosan (CMC) NPs have been loaded with a very potent AMP OH30 extracted from king cobra for potential wound healing. Surprisingly, positively charged CMC could interact with negatively charged bacterial cell membranes and assist the internalization of the OH30 peptide. A slow release of OH30 from the CMC-OH30 NPs was seen over 24 h and maintaining antimicrobial activity as well. CMC-OH30 NPs could slightly enhance migration (95%) but not the proliferation of keratinocytes compared to OH30 (85%), CMC NPs (75%), or untreated cells (60%). In vivo, CMC-OH30 NPs significantly accelerated wound healing in a full-thickness excision mouse model. Moreover, mice treated with CMC-OH30 NPs exhibited 70% wound closure at day 5 compared to CMC NPs or OH30 alone, which maintained wound closure in the range of 36–58% [150]. A drawback of the CMC-OH30 complex is its limited stability as shown by the variability in size and zeta potential over 28 days. Another 5-amino acid AMP, RBRBR, was encapsulated into chitosan NPs through the ionotropic gelation process with 51% encapsulation efficiency, slowly releasing the AMP over 14 days. AMP-loaded chitosan NPs showed at least 3-log increased antimicrobial activity against *S. aureus* while decreasing the toxicity against both mammalian and human erythrocyte cells. Importantly, positively charged (+33 mV) AMP–chitosan NPs (121 nm) significantly inhibited the growth of biofilm (up to 98%) [151]. The straightforward approach of ionotropic gelation may be used to deliver other potent AMPs while reducing their cytotoxicity, in order to achieve efficient antibacterial effects against MDR and biofilm-forming bacteria. 

Nordström et al. investigated the ability of poly(ethyl acrylate-*co*-methacrylic acid) (MAA) microgels to deliver two AMPs, LL-37 and DPK-060. AMP loading into the microgels decreased toxicity toward erythrocytes and was suggested to protect peptides against protease degradation. In turn, microgel-loaded peptides could kill MRSA, *E. coli*, and *P. aeruginosa* via a membrane disruption mechanism [152]. Further, in an effort to elucidate the mechanism of interaction between microgel-loaded AMPs and cellular membranes, Nordström et al. included LL-37 into MAA microgels. The insertion of the peptides released from LL-37-loaded microgels was shown to occur without membrane defect formation at low concentrations, but with the loss of lipid from the bilayer in a concentration-dependent manner [153]. Similarly, LL-37 has been encapsulated into liquid crystalline nanoparticles (LCNPs), such as cubosomes, for the treatment of *S. aureus* skin infection. LL-37 loaded into cubosomes was protected against proteolytic degradation by *P. aeruginosa elastase* while improving AMP bioavailability and efficiency. In an ex vivo wound infection model, LL-37-loaded cubosomes showed the highest killing efficiency of *S. aureus* and an absence of pig skin irritation [154]. Further molecular dynamic simulations confirmed these experimental results, revealing that charged amine and guanidium groups from LL-37 are responsible for facilitating the interaction with the bacterial membrane. Moreover, LL-37 stabilized the cubosome through hydrophobic interactions, while polar residues remained in solution [155].

Silva et al. loaded LLKKK18 (KEFKRIVKRIKKFLRKLV) into hyaluronic acid nanogels and reported a higher stability of the peptide and reduced toxicity to mammalian cells. Importantly, it was found that nanogels were internalized by macrophages infected with *Mycobacterium tuberculosis* or *M. avium*. A significant reduction of bacterial load in infected mice after tracheal administration of the nanogels was seen [156]. Similarly, tet213 AMP was admixed to alginate, hyaluronic acid, and collagen for a potential wound dressing. Tet213 dressings showed antimicrobial activity against three different bacterial strains (*E. coli*, MRSA, and *S. aureus*) and promoted fibroblast proliferation in vitro. Moreover, in vivo Tet213 dressings promoted re-epithelialization, enhanced collagen deposition, angiogenesis, and the completion of wound healing in *E. coli*- and *S. aureus*-infected full-thickness wounds in rats. The AMP dressing had higher antibacterial activity than silver-containing commercial Aquacel, alginate, hyaluronic acid, and collagen dressing [157]. 

**Figure 7:**
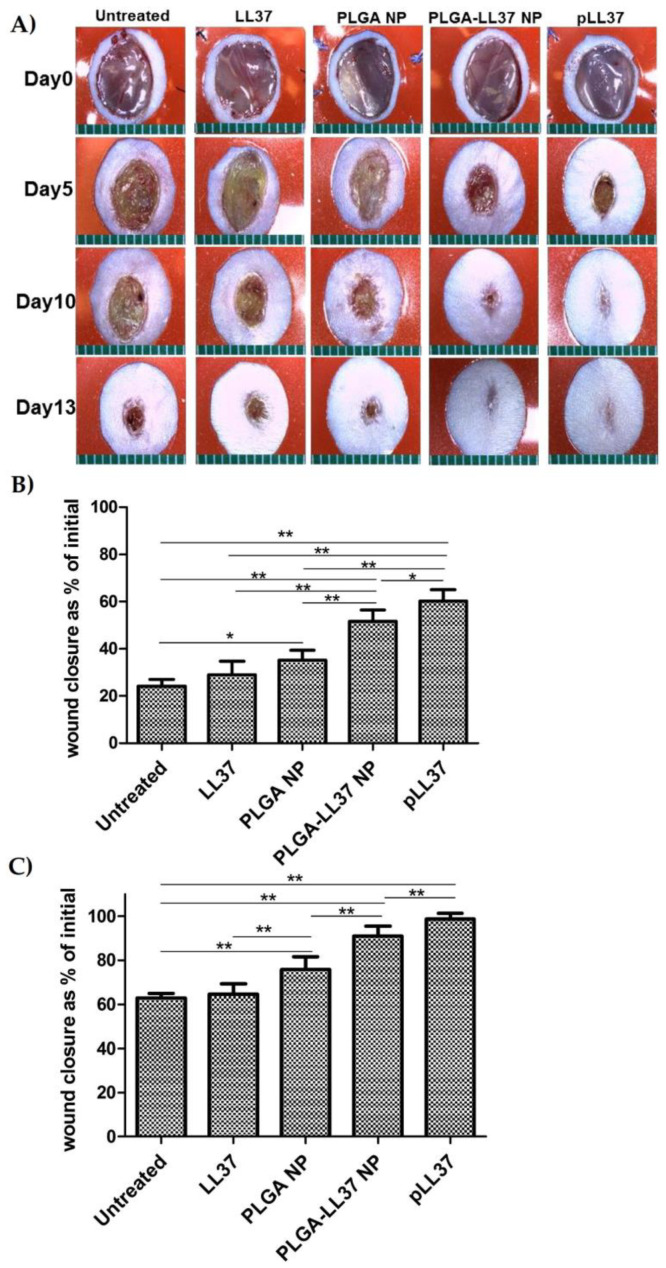
Accelerated wound healing in mice treated with PLGA-LL-37 nanoparticles (NPs) compared to controls. (**A**) Images of wounds in mice of five tested groups: untreated, LL-37, PLGA-NP, PLGA-LL-37 NPs, and pLL-37 (plasmid encoding hCAP18/LL-37). Representation of wound area at (**B**) day 5 (*n* = 13) and (**C**) day 10 (*n* = 10) (mean ± SD). Values of *p* < 0.05 * and *p* < 0.01 ** were indicative of statistically significant differences. Reprinted from [158] with the permission of Elsevier©, Copyright 2014.

As for solid micro/nanoparticles, LL-37-loaded PLGA NPs were prepared via a water-in-oil-in water (W/O/W) emulsion–solvent evaporation method and showed high antibacterial activity in vitro against *E. coli*. Topically applied PLGA-LL-37 NPs enhanced wound closure in mouse full-thickness excisional wounds with an average healing of 79% and 90% at days 7 and 10, respectively (Figure 7). Moreover, advanced granulation tissue formation, re-epithelialization, and accelerated angiogenesis was observed in the group of mice treated with PLGA-LL-37 NPs [158]. The only disadvantage of such system is the limited residence time and bioadhesion in the wounds, which is not desired in the case of hard-to-heal wounds. Similarly, esculin1-a AMP loaded into PLGA NPs resulted in selective bioactivity with high antibacterial activity against *P. aeruginosa* in both in vitro and in vivo and decreased cytotoxicity. Despite that AMP loaded NPs showed lower antibacterial activity in vitro than free esculin1-a at 24 h, they showed a constant inhibition of *P. aeruginosa* growth up to 72 h. Furthermore, AMP-loaded NPs (0.1 mg/kg) in a mouse model of acute *P. aeruginosa* lung infection were shown to significantly reduce the bacterial load by 17-fold compared to free esculin1-a in solution [159].

The performance of AMP incorporated into micelles was investigated by Wang et al. After complexation process between MSI-78 (pexiganan) peptide and methoxy poly(ethylene glycol)-*b*-poly(α-glutamic acid) (mPEG-*b*-PGlu), it was found that hemolytic toxicity was decreased through polyelectrolyte complexation without hindering its antimicrobial activity against *E. coli*, *B. subtilis*, and *S. aureus* [160]. In another study, peptide 73c and D-73 encapsulated into PEGylated micelles showed increased antibacterial activity against *S. aureus* biofilms by 510- and 9-fold, respectively, compared to their parent peptide in a murine cutaneous abscess model. In vitro, peptides formulated as PEGylated micelles exhibited decreased toxicity to mammalian cells [94]. 

LL-37-loaded nanostructured lipid carriers (NLC) were prepared by the melt-emulsification method and showed a preserved bioactivity against *E. coli* (killing efficiency 73%). Topically applied NLC-LL-37 to the wounds enhanced wound closure, re-epithelization, collagen deposition, and angiogenesis in diabetic mice [161]. The major drawback of such a system is the limited residence time in the wounds.

#### 4.2.2. Liposomes

Liposomes as drug carriers have various advantages for providing sustained and controlled drug release for topical applications [162]. Examples of commercially available liposome-based antibiotics that encapsulate amphotericin antibiotic include AmBisome^®^, Fungisome^®^, and Amphotec^®^ and lipid-based Abelcet^®^ [163]. 

In this view, vancomycin was used as a model drug by Yang et al. [164]. Drug-loaded liposomes were coated with chitosan in order to improve vancomycin pharmacokinetics and exhibited sustained drug release both in vitro and in vivo, without inducing hemolysis. Importantly, chitosan-coated vancomycin-loaded liposomes had low concentration in kidneys and high concentration in lungs compared to free vancomycin or vancomycin-loaded liposomes without chitosan coating. Such a system could reduce nephrotoxicity caused by free vancomycin, while enhancing the effect in lung infection. Similarly, nisin encapsulated into cationic liposomes exhibited efficient microbicidal activity against *S. mutans* [165]. 

Polymyxin B has been encapsulated into liposomes to target Gram (−) bacterial infections in the sputum of cystic fibrosis patients. Immunocytochemistry revealed a high cell uptake of polymyxin-loaded liposomes, leading to high antimicrobial efficiency toward *P. aeruginosa*. The bioactivity of liposomal polymyxin B formulation was 4-fold higher compared to AMP alone [166]. Similarly, He et al. encapsulated polymyxin B in a liposomal formulation prepared via the reversed-phase evaporation method. IV injection of liposomal polymyxin exhibited an enhanced pharmacokinetic profile of polymyxin in a pneumonia mice model. Moreover, the efficacy of IV liposomal polymyxin B was significantly higher against *P. aeruginosa* compared to naked liposomes or polymyxin in solution [167]. The limitations of these studies are the absence of toxicity and hemolysis data, which are critical when polymyxin B is involved. 

Still, the success of liposomes depends on understanding the interactions between liposomes and the target site. In this view, Li et al. investigated daptomycin-loaded liposomes for topical skin infections. Both pharmacokinetics and pharmacodynamics were enhanced when using optimal lipid ratios of lecithin to sodium cholate 17:1 (*w*/*w*) and a lipid-to-drug ratio of 14:1 (*w*/*w*). The liposomal formulation could permeate the skin efficiently and successfully deliver AMP to the site of infection inhibiting *S. aureus* in mice [168]. 

Overall, the liposome vectorization offers interesting features for the modulation of pharmacokinetics, which could warrant further investigations for AMP delivery. 

### 4.3. Inorganic NPs

#### 4.3.1. Metallic NPs

The two most used systems of metal NPs are gold (AuNPs) and silver (AgNPs) due to their broad-spectrum activities [169]. These systems offer suitable strategies for delivering AMPs, as both counterparts are usually of low molecular weight. In addition, due to their high surface-to-volume ratio, AMPs can be easily adsorbed to the surface of NPs, resulting in a relatively high AMP loading and efficient delivery to eradicate bacteria. Within this context, esculentin-1a after covalent coupling to AuNPs via a PEG linker showed a 15-fold increased antibacterial activity against *P. aeruginosa* while being non-toxic to keratinocytes in vitro. Au/esculentin-1 NPs were more resistant to proteolytic degradation with higher potency against *P. aeruginosa* than the parent peptide and displayed wound-healing properties on keratinocytes in vitro [170]. AMP-coated AuNPs were the first NP system to be proposed for topical application providing antibacterial and wound-healing properties. In another study, AuNPs functionalized with indolicidin were obtained through thiol chemistry by covalently attaching AMP and to AuNPs. The Au/indolicidin nanosystem presented higher efficacy in preventing *C. albicans* adhesion and eradicated biofilm formation in vitro, as compared to indolicidin alone [171]. The enhanced activity of indolicidin was attributed to the protection from protease degradation conferred by the incorporation into the NPs. In an in vivo study, two AMPs (cecropin and melittin) were conjugated to AuNPs and tested in a sepsis *P. aeruginosa*- and *S. aureus*-infected mouse model. AMP–AuNPs (200 µL; 5 mg/mL) lead to decreased bacteremia and significantly lower systemic inflammation than the free peptides. The nanosystem showed higher efficiency than the free peptides in vitro against *S. aureus*, *E. coli*, and *P. aeruginosa*, while being non-hemolytic, non-cytotoxic, and non-immunogenic [172]. 

A D2A21 AMP encapsulated into Ag nanocomposite exhibited significantly increased antibacterial potency compared to native AMP and AgNPs (MIC: 1 to 3 μg/mL versus 4–6 μg/mL) against *P. aeruginosa*, *S. aureus*, and *E. coli* via membrane damage [60]. An Andersonin-Y1 peptide has been conjugated to AgNPs through a peptide containing a cysteine linker either at the N- or at the C-terminal. Both systems resulted in high activity against *K. pneumonia*, *P. aeruginosa*, and *Salmonella typhi* (*Enterbacter* spp.), which was nearly 10-fold higher than the parent peptide [140]. The activity of AgNPs against MDR bacteria was enhanced when assisted by the AMP, which disrupted the bacterial membrane, allowing AgNPs to kill bacteria. In another study, the odorranain-A-OA1 (OA1) peptide was coupled to AgNPs through a cysteine in order to stabilize to the nanosystem. AgNP–OA1 conjugates exhibited enhanced bacteria leakage in *E. coli* and reduced cytotoxicity [173]. Lambadi et al. investigated AgNPs conjugated to polymyxin B and found polymyxin B-AgNPs to display at least 3-fold higher bacterial killing than AgNPs alone. Likewise, AMP–AgNPs showed high potential in destroying biofilm in MDR *Vibrio fluvialis* and *P. aeruginosa* [174]. Similarly, Zheng et al. studied Ag nanoclusters conjugated to daptomycin for their antibacterial activity against *S. aureus*. The nanocluster–AMP system showed higher antibacterial efficiency than the simple mix of daptomycin and Ag nanoclusters. It was suggested that higher antibacterial activity was due to the localization of Ag nanoclusters into the core of the hybrid structure, which could generate reactive oxygen species (ROS) and lead to cell death [175]. 

Altogether, AMP–metal nanocomposites resulted in synergistic antibacterial effects with enhanced antibacterial activity at low cytotoxicity. Moreover, they proved to be efficient in eradicating biofilms, as they can disrupt the bacterial membrane and finally lead to lysis. However, these nanosystems contain Au or Ag, which are known to be poorly biocompatible and non-biodegradable over long periods of time, and their elimination from the human body is not completely understood [3,176]. AgNPs compounds show very promising results in vitro, although their application can be hindered due to their toxicity seen in vivo [69,72]. As for Au, very recently, Balfourier et al. investigated AuNPs and concluded that Au can be metabolized by mammalian cells. They studied the biotransformation of AuNPs (4 to 22 nm) in primary human fibroblasts for 6 months. First, they could observe that AuNPs follow intracellular degradation induced by ROS that oxidize AuNPs in a size-dependent manner. Second, they demonstrated that gold undergoes a biomineralization process and recrystallizes as nanoleaves (2.5 nm) or so-called aurosomes [177]. Therefore, this brings new insights into the lifecycle of AuNPs and their elimination from the organism.

#### 4.3.2. Carbon Nanotubes, Graphene, Fullerenes

The latest studies in carbon-based nanomaterials, such as graphene, fullerene, carbon quantum dots (CQDs), and carbon nanotubes (CNT) have attracted increasing interest due to their potency in combating infections, while some reports suggest an acceptable toxicity toward mammalian cells and tissues [178,179]. In addition to their antimicrobial potency, carbon-based nanomaterials are very attractive for drug delivery [43]. For instance, CQDs coupled through pyrolysis to L-lysine (Lys-CQDs) and L-arginine (Arg-CQDs) showed effective and selective antibacterial potency with no resistance against *E. coli* and *S. aureus*. The in vitro efficacy of the system was due to the induction of intracellular ROS by CQDs. However, no cytotoxicity was reported in mammalian cells. On the contrary, Lys-CQDs and Arg-CQDs promoted the growth of mammalian cells and no hemolysis to red blood cells in vitro. In vivo, both systems significantly inhibited bacterial growth and accelerated wound healing in infected wounds in mice [178]. These systems could lead to different clinical applications of CQDs to eradicate bacteria and promote tissue repair. 

Carbon nanotubes (CNTs) are formed by rolled-up tubular shells of graphene. Chaudhary et al. studied the bioconjugation of silver-coated CNTs to AMP TP359 for their antimicrobial potency against MDR Gram (+) (*S. aureus* and *Streptococcus pyogenes*) and Gram (−) (*Salmonella enterica serovar Typhimurium* and *Escherichia coli*). The bioconjugated system exhibited synergistic antibacterial effects of TP359 and Ag-CNTs and minimal toxicity toward murine macrophages and Hep2 cells within their MIC range [180]. Indolicidin conjugated to CNT and AuNPs exhibited an enhanced potency of indolicidin in an in vitro study. CNT–indolicidin conjugates were found to preserve the activity of indolicidin at a 1000-fold lower concentration. The CNT-carrier activated more pro-inflammatory genes, while AuNP conjugates stimulated the expression of the anti-inflammatory IL-10 gene [181].

Nisin was conjugated to a 3D graphene membrane for the removal of MRSA from water. The graphene system was shown to trap and kill all the bacteria (close to 100%) using a nisin-conjugated membrane. Moreover, the nisin–graphene membrane showed synergistic effects compared to graphene oxide alone or to nisin alone. This time, the multimodal mechanism of action could be observed. Bacteria were captured into the graphene’s foam, which caused stress to the bacterial membrane, allowing nisin to enter, bind easier to bacteria, and kill them efficiently [182]. In addition, Nellore et al. coupled a PGLa (GMASKAGAIAGKIAKVALKAL-NH_2_) AMP and CNT with a hybrid membrane of glutathione-conjugated carbon nanotube (CNT) bridged three-dimensional (3D) porous graphene oxide for the efficient removal of *E. coli*. It was found that the system was not only able to capture the bacteria from water, but also to kill *E. coli* efficiently [183].

A few studies proved that AMPs could be integrated into fullerene C_60_ nanocrystals using C_60_ carboxylic groups, which enables covalent coupling between fullerenes and AMPs. Maximin H_5_ peptide mutants (extracted from Asian toads) were conjugated to fullerenes and showed higher antiviral activities than the maximin H5 peptide [184]. Baron demonstrated that fullerene–peptide conjugates could facilitate transdermal transport in an ex vivo porcine model, whereas the parent peptide did not show any permeation. The fullerene–peptide system could dramatically modify cellular uptake and showed less toxicity to keratinocytes at concentrations below 0.04 mg/mL. They exhibited high efficacy against *S. aureus* but were inactive against *E. coli* and *C. albicans* [185]. Furthermore, AMP–fullerene systems were developed with high bacteriostatic activity against *S. aureus* and *E. coli*, exhibiting low MICs of 8 and 64 μM, respectively [186].

Altogether, carbon-based nanosystems show promising results for AMP delivery. Still, concerns of toxicity, induction of granuloma, and potential carcinogenicity in specific models warrant extensive safety studies encompassing a thorough evaluation of their long-term toxicities and pharmacokinetics properties [185,187]. 

### 4.4. Smart Nanomaterials

Smart nanomaterials are inspired by natural mechanisms such as those observed in cephalopods (i.e., squid, cuttlefish, or octopus) that respond to external stimuli by changing their color. These so-called “biomimetic” materials can modify their properties in response to external chemical stimuli (e.g., pH, ionic strength) via different interactions. They may offer opportunities to deliver drugs to a specific site or target with an enhanced biological activity. 

**Figure 8:**
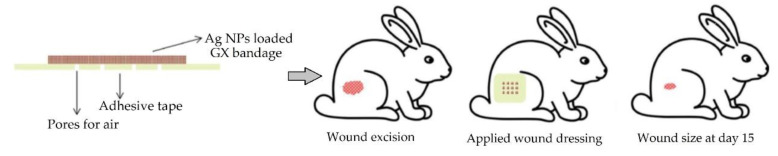
Preparation of smart wound dressing based on AgNPs–GX (gold nanoparticles–glucuronoxylan) hydrogels and their application for wound treatment. Modified from [190].

Hydrogel-forming polysaccharides are widely used in pharmaceutics and cosmetics. They have been used as capping agents for the formulation of AgNPs. Glucuronoxylan (GX) has been used due to its pH-responsive swelling properties upon shifting of the pH from acidic to neutral [188]. This was intended to follow the natural wound pH variation from acidic to slightly alkaline during the different phases of wound healing, due to the imbalance between tissue remodeling and degradation processes [189]. Another antibiotic, fluconazole, has been successfully incorporated into GX hydrogels for drug delivery applications [188]. In another study, AgNPs were prepared using GX polysaccharide to deliver Ag to the infection site. Both GX AgNPs (50 mmol, 5 h sunlight exposure) and Band aid^®^ patches showed comparable wound-healing abilities [190]. The high moisturizing effect of hydrogels at neutral pH is highly beneficial for patients, preventing bandage adhesion. Moreover, incorporated AgNPs showed high bactericidal and fungicidal activity against *S. epidermidis*, *E. coli*, *P. aeruginosa*, *B. subtilis*, *S. aureus* and *A. niger*, *P. notatum*, *R. stolonifer*, and *A. odontolyticus*. As well, rapid wound-healing tendency was seen when using AgNPs–GX hydrogels in rabbits (Figure 8). 

Electrospun hybrid polyacrilonitrile (PAN) nanofiber mats have been developed for wound healing, possessing the ability to monitor in real-time the H_2_O_2_ level in the wound [191]. High H_2_O_2_ concentration in wounds leads to delayed healing, hindering connective tissue formation [192]. The PAN smart dressings could sense H_2_O_2_ concentration through fluorescence changes upon UV irradiation, indicating the level of bacterial infection and the time to change the mats. Such smart dressing approaches could help monitor in vivo wounds in an inflammatory model. Real-time information could help avoid infection and shorten the healing time.

Nanofibrous electrospun membranes were prepared by loading an antibiotic, such as nitrofurazone, into Eudragit^®^ S100 (ES 100) polymer solution as potential smart nanomaterial for skin infection treatment. ES100 is a pH-responsive polymer that dissolves at pH higher than 7, thus triggering the release of the antibacterial agent [189]. Rezaei et al. have prepared thermo-responsive chitosan (TCTS) hydrogels and loaded them with piscidin-1 AMP at 16 μg/mL to obtain efficient antibacterial wound dressing against resistant strains of *A. baumannii*. The use of TCTS hydrogels as a carrier for piscidin-1 showed to be advantageous with high antibacterial activity and an absence of toxicity to mammal cells [193].

**Figure 9:**
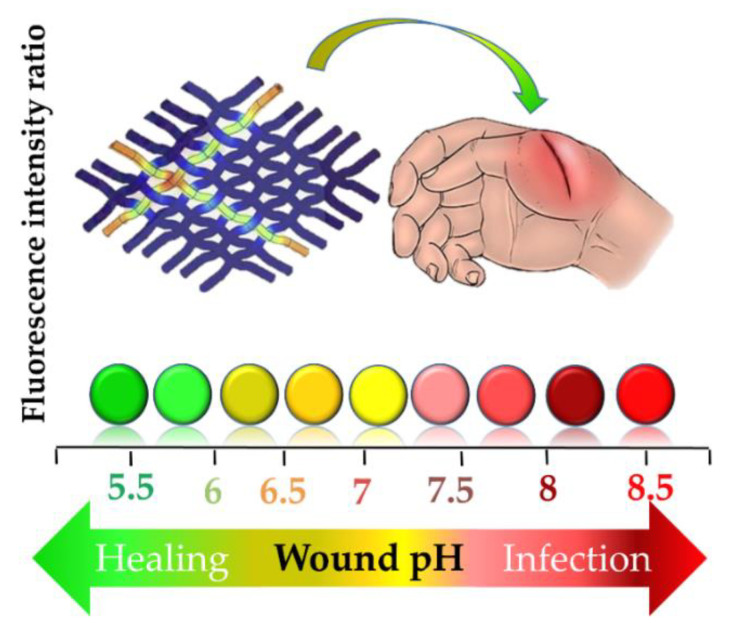
Understanding wound status: elevated or extremely low pH in wound bed can be a sign of infection. Modified from [194].

Panzaras et al. [194] developed “bandages with a voice” using a pyranine–benzalkonium ion pair loaded into wound dressings that are able to detect pH changes in the range of 5.5–7.5, based on the UV-induced fluorescence color change of a dye. The pH of the healing wound is around pH 5–6, while the pH in chronic wounds fluctuates between 7 and 8, indicating bacterial infection (Figure 9). These kinds of dressings are practical and very much needed for monitoring the status of the wounds, although the pH sensitivity range is quite narrow, and a UV lamp is required. 

A very sound strategy to design smart nanomaterials has been proposed by Lombardi et al. [195]. Their system could incorporate conventional antibiotics while being stimuli responsive, so at a certain pH, it would release the antibiotic. An AMP developed from innate immunity peptides found in the hagfish, WMR, was linked to an aliphatic residue having a lipidic tail with pH-dependent properties. For instance, at pH 10, the peptides self-assemble into stable nanofibers that are highly active against *C. albicans*, *P. aeruginosa*, and biofilm bacteria, while at pH 3 and 7, they do not self-assemble. These nanostructures hold great potential as stimuli-responsive materials for infection treatment in the wounds. 

RADA16, a self-assembling peptide that is able to form 3D-hydrogel in response to pH changes, has received considerable attention due to its remarkable properties to deliver different proteins and drugs, to promote tissue regeneration, and to influence cell behavior [196]. It is worth noting that proteins can preserve their original structure and function inside RADA16 hydrogels. RADA16 was functionalized with a RGDS fibronectin moiety (RADA16-RGD) or with a FPGERGVEGPGP collagen-1 (RADA16-FGP) motif via two glycine residues to ensure flexibility to the functional groups. Functionalized RADA16-FGP was shown to promote the migration of keratinocytes and fibroblasts significantly, while RADA16-RGD had no effect on cells [197]. In addition, RADA16-RGD added with TGF-β1 was used for growing bone mesenchymal cells from rats over the course of 2 weeks. The results showed that RADA16 peptide hydrogels were able to release active TGF-β1 growth factor and significantly influence the proliferation of stem cells [198].

These types of smart biomaterials could induce diverse cell responses within a regenerative scaffold. Therefore, the incorporation of AMP in such smart and wound environment-responsive systems could be successfully used to promote not only wound healing but to protect wounds against microbial infections. 

Overall, incorporating AMP into different delivery nanosystems showed an improved bioactivity with higher rate of bacterial killing while reducing their toxicity. Very surprisingly, Håkansson et al. loaded DPK-060 into three different nanosystems: lipid nanocapsules (LNCs), nanocubes, and polaxamer gels compared to the parent DPK-060 peptide in acetate buffer. No enhanced effect was found when encapsulating DPK-060 in any of the formulations, except when including AMP in polaxamer gels [199]. This points out the need for a specific optimization of the carrier for a given peptide through activity and toxicity testing. 

## 5. Discussion and Further Insights

AMPs have raised attention due to their potent activity along with many advantages, such as broad activity not only against common strains, but MDR bacteria as well, which is of utmost interest nowadays. They show relatively low bacterial resistance and high activity against biofilm-forming bacteria, especially critical in implant- or device-related infections, although very little is yet known about resistance mechanisms developed by biofilms. 

The main limitations for the use of AMPs are summarized in Table 3. Poor stability may be overcome by several approaches, as mentioned in Section 3. Cytotoxicity and hemolysis are critical issues that need to be addressed, for instance by conjugation, PEGylation, or formulation as detailed above. Finally, production costs might be an additional barrier to the clinical use of AMPs. 

Clinical research on AMPs may bring insight into future developments. Only a minority of AMPs has obtained FDA approval. Neosporin^®^ (gramicidin), Cubicin^®^ (daptomycin), Vancocin^®^ HCl (vancomycin), Orbactiv^®^ (oritavancin), and Dalvance™ (dalbavancin) have been approved for the treatment of bacterial skin infections or wounds [44]. Most of the currently approved AMPs, except colistin, are used to treat Gram (+) bacteria, and a few of them have low oral bioavailability or poor intestinal mucosa penetration and fast enzyme degradation [200]. On the other hand, oral Vancomycin^®^ HCl administration in patients with severe colitis and renal insufficiency leads to severe intestine mucosal damage due to extremely high bioavailability [201]. Therefore, drug monitoring is highly suggested in cases of high risk, such as colitis or renal insufficiency. Moreover, AMPs that are efficient to treat Gram (−) bacteria are now emerging.

Although vancomycin has been FDA approved and used to treat MRSA- and *C. difficile*-colitis, it has been found to induce peripheral rash, anaphylaxis, “red man syndrome”, ototoxicity, and nephrotoxicity [201,202]. Daptomycin is used as an alternative to vancomycin and has been FDA approved for MRSA bloodstream infections. On the other side, colistin and daptomycin are considered as extremely important AMPs for the treatment of severe human diseases and other diseases transmissible from non-human sources to humans [203]. However, colistin may cause severe neuro- and nephrotoxicity and is used only as a “last-resort” antibiotic [203]. Telavancin, another derivative of vancomycin, has been approved for MDR infections, especially for osteomyelitis treatment, but it leads to adverse effects, such as nausea, vomiting, diarrhea, and metallic taste [204]. Oritavancin has fewer side effects than vancomycin and was found to be safe in patients with osteomyelitis even when administering multiple doses a day [205]. These clinical outcomes suggest that it is essential to check the selectivity when designing a new AMP with increased bioactivity and reduced toxicity. Progress in AMP design, testing, and vectorization suggests that the number of AMPs will be increasing in the near future to combat MDR bacteria in wound infections and biofilm-related infections. 

Toxicity to mammalian cells and AMP short half-life are main factors limiting their clinical application. Therefore, the topical delivery of AMPs, especially to the wounds, is the most common route of administration. Still, AMPs could be easily degraded by wound exudates. Therefore, the different delivery strategies described herein, such as conjugation of the AMPs to polymers, encapsulation into different nanosystems, and smart nanomaterials or new technologies such as CRISPR-Cas9 could be used to overcome the hurdles of AMPs alone while maintaining high antimicrobial activity. So far, there is no commercial formulation available specifically for the topical application of AMPs. 

Advanced gene engineering tools for AMP synthesis, such as Clustered Regularly Interspaced Short Palindromic Repeat (CRISPR)-associated protein (CRISPR-Cas, a RNA-guided genome editing system) and Zinc-Finger Nucleases (ZNFs), are new approaches recently exploited that are still not fully understood but deserve future research and investigations [206]. Such modern tools can be used to specifically target AMR bacterial strains that carry those resistant genes and re-sensitize bacteria to antibiotic treatment [207]. For example, the group of Lemaitre has been using the CRISPR-Cas9 endonuclease to delete 14 AMP genes of *Drosophila*. The results showed that the obtained AMP mutants were efficient against Gram-negative bacteria (e.g., *E. coli* and Enterobacter species) and fungi in *Drosophila* [208]. The same group could observe that only *Diptericins* could resist the infection of *Providencia rettgeri* [208], which may correlate with the polymorphism in *Drosophila* [209]. CRISPR-Cas9 mediated AMP gene knockout could serve as an example of how to engineer novel and efficient AMPs for patients lacking a specific AMP to prevent certain infections. 

Choudhary et al. 2019 [210] and Wei et al. 2019 [211] have used the endogenous CRISPR-Cas system as an efficient tool against *Mycobacterium tuberculosis*, which has developed multidrug-resistance, even against the most potent antibiotic rifampicin. In other studies, CRISPR-Cas9 was successfully delivered to selectively kill MDR bacteria *E. coli* [212], and *S. aureus* [213] using a phagemid (plasmid packed in a phage capsid)-mediated system by removing plasmids carrying AMR genes and, in turn, effectively re-sensitizing bacteria to antibiotics. Moreover, both the phagemid-mediated delivery of CRISPR-Cas9 was shown to be highly effective in vivo in *E. coli*-infected *Galleria mellonella* larvae [212] and *S. aureus* in a mouse skin colonization model [213].

Although CRISPR-Cas proved to be an efficient tool to enhance AMP antimicrobial activity as antimicrobials, it is very important that the protospacer adjacent motif (PAM) sequences and Cas proteins are fully characterized [214]. In addition, the use of recombinant microbial platforms for genetically tailoring AMPs leads very often to low yield and poor quality of the final product [206]. Therefore, a very limited number of genetically modified AMPs [215] have reached clinical trials; thus, these AMPs are less marketed, which has been described in detail by Kosikowska and Lesner.

With respect to delivery strategies, general trends may be distinguished despite the lack of comparative studies in the field. A salient feature is the ability of several delivery strategies to protect the peptide from degradation and/or to sustain its activity at the diseased site. This is true for several polymer conjugates, but also for polymer nanogels. Chitosan stands out as a promising biopolymer in this respect. Nanotechnological platforms may also be used to improve solubility and bioavailability. As a final topical formulation, these may be integrated into a polymer scaffold to provide both controlled peptide delivery and additionally promote wound healing.

Still, the safety of these systems needs careful assessment, which should include their hemolytic activity and potential side effects. In this respect, more recent approaches relying on metallic nanoparticles, nanotubes, or fullerenes warrant further investigation to confirm a beneficial efficacy/toxicity balance.

Finally, combined strategies, such as the use of antibiotics for synergistic action or the combination with antibiotic resistance breakers might be required to further potentialize anti-infective therapy.

Altogether, it is important to design new AMPs with high antibacterial activity, but it is essential to optimize them via proposed strategies to efficiently and safely deliver them at the site of infection. AMPs remain one of the most promising classes of antimicrobial agents that brings hope to overcome AMR and biofilm-related infections.

## Figures and Tables

**Table 1:** AMPs with their potential activity selected from the Antimicrobial Peptide Database (APD) [20].

Activity AMP	Selected Examples of AMPs	Total No.
Antibacterial peptides	Abaecin; andropin; bombinin; ^1^ bBD-1-13; cecropin A, B, C, D, P; cryptdin; drosocin; esculentin-1-2; dermaseptin-B2-B5, B6, S1-S4; ^2^ hBD-26,27; LL-37; magainin; melittin; nisin; protegrin 1; pyrrhocoricin; temporin A, B, C, E, F, G, K, L; thanatin; tritrpticin	2678
Antibiofilm peptides	BMAP-27,28; citropin 1.1; colistin A; Dhvar4; gramicidin S; hBD-3; holothuroidin 1; indolicidin; LL-37; nisin A; polymyxin B; protegrin 1; SMAP-29 (Ovispirin); tachyplesin III; temporin B; temporin-1CEb	57
Anticancer peptides	Alloferon 1,2; aurein 1–3; buforin II; gomesin; indolicidin; lactoferricin B; LL-37; magainin 2; mastoparan B; melittin; nisin A,Z; tritrpticin	237
Anti-diabetic peptides	Amolopin; brevinin-1E, 2EC; esculentin-1, 1B; magainin-AM2	15
Antifungal peptides	Androctonin; antifungal protein; aurein 1–3; cecropin 2, A, B; dermaseptin-S1-S5; HD-2-6; HNP-1-6; indolicidin; lactoferricin B; magainin 2; melittin; protegrin 1–5; ponericin G1-G4; G7, W1-W5; thanatin; tritrpticin	1142
Anti-HIV peptides	Aurein 1.2; cecropin A; dermaseptin-S1,S4, S9; hBD-2,3; HNP-1-4; indolicidin; lactoferricin B; LL-37; melittin; protegrin 1	109
Anti-inflammatory peptides	Allomyrinasin; cathelicidin-PY; coprisin; defensin DEFB126; lucilin; papiliocin	20
Anti-MRSA peptides	Acipensin 1,2; BMAP-27,28; CAP18, citropin 1.1; clavanin A; cryptdin-4; ^4^ Dhvar5; esculentin-1,2 ISa-ISb; hBD-3; hedistin; ^3^ HNP-1; hominicin; imcroporin; indolicidin; LL-37; micasin-1; omega76; SMAP-29; plectasin; pleurocidin; protegrin 1; ubiquicidin	165
Antiparasitic peptides	Batroxicidin; cecropin A; dermaseptin-S1-S5; kalata B2, B5-B7; LL-37; magainin 2; melittin; temporin A, B, F, L;	116
Anti-sepsis peptides	Apidaecin IA; bactenecin 7; buforin II; cathelicidin-PY; cecropin 2, P1; drosocin; ^5^ HD-5; HNP-1; lactoferricin B; LL-37; melittin; polymyxin B; protegrin 1; pyrrhocoricin; SMAP-29; tachyplesin I; temporin L; thanatin	75
Anti-toxin peptides	hBD-1-4; HNP-1-5; retrocyclin-1-3	15
Anti-tuberculosis peptides	Griselimycin; hBD consensus; hBD10; human granulysin; lassomycin; laterosporulin10; LL-37; micrococcin P1; pantocin wh-1; RNase 7; Teixobactin; VpAmp1.0, 2.0	13
Antiviral peptides	Alloferon 1,2; antiviral protein Y3; aurein 1.2; BMAP-27,28; dermaseptin-S1, S4; hBD-1-3; HNP-1-6; indolicidin; lactoferricin B; LL-37; magainin 2; melittin; mucroporin; protegrin 1–5; thanatin; temporin A, B	189
Wound-healing peptides	AH90; AG-30; AG-30/5C; bactenecin; coprisin; epinecidin-1; hBD-2, 3; HD-5; HNP-1; IDR-1018; indolicidin; LL-37; lucifensin; magainin 2; nisin A; temporin A	22

^1^ bBD: bovine beta defensin; ^2^ hBD: human beta-defensin; ^3^ HNP: human neutrophil peptide; ^4^ Dhvar: human Histatin; ^5^ HD: human defensin.

**Table 2:** Selected AMPs for topical application under different phases of preclinical and clinical trials, including AMPs FDA-approved and some potent AMPs based on their reported activities.

AMPs	Mechanism of Action	Activity Against	Side Effects	Application and Administration Route	Ref
**Potent AMPs**					
AG30	Membrane disruption	Gram (+), (−); *E. coli* (MIC: 40 µg/mL) and *S. aureus* (MIC: 20 µg/mL)	Lack of stability in saline	Topical	[36]
AG30/5C	Membrane disruption	Gram (+), (−); *P. aeruginosa* (MIC: 5 µg/mL), *S. aureus* (MIC: 50 µg/mL)	More stable than AG30	Topical	[37]
Cys-KR12	Membrane disruption	Gram (+), (−); *E. coli* (MIC: 4 µg/mL), *S. aureus* (MIC: 8 µg/mL)	Not reported	Topical	[45]
KR12	Membrane disruption	Gram (+), (−); *E. coli* (MIC: 2.5 µM), *P. aeruginosa* (MIC: 10 µM)	Not reported	Topical	[46]
WLBU2	Binding to lipopolysaccharide and DNA inhibition	Gram (+), (−); *P. aeruginosa* (MIC: 3–8 µM); *S. aureus* (MIC: 6–16 µM)	Not reported	Eradicated lethal *P. aeruginosa* septicemia in mice	[47,48]
WRL3	Membrane lysis	Gram (+), (−); MRSA (MIC: 2 μg/mL)	Not reported	MRSA-related infections in skin burn wounds; topical	[41]
**Preclinical Phase**					
Arenicin	Membrane pore formation	Gram (+), (−); MRSA infection; *E. coli* (MIC: 1 µg/mL); *K. pneumoniae* (2 µg/mL)	Significant toxicity to mammalian cells	Urinary tract infections; hospital-acquired infections	[23,49,50]
Avidocin and purocin	Membrane disruption	Gram (+) and (−)	Safety reported	Treatment of *C. difficile* infections (colitis); topical	[23]
Buforin II	Inhibition of DNA/RNA synthesis	Gram (+), (−); fungi; *E. coli* (MIC: 1 µg/mL); *K. pneumoniae* (MIC: 2 µg/mL)	Safety reported	Used as bacteriostatic; bactericide; anti-sepsis	[25,51]
Lactocin 160 (Bacteriocin)	Membrane disruption	*G. vaginalis*; *P. bivia*; *Lactobacillus* spp. (MIC: > 200 mg/mL)	Safety reported	Urogenital tract infections; Bacterial vaginosis	[52,53]
LTX-109 (Lytixar)	Membrane disruption and cell lysis	Gram (+); MRSA; ^2^ VISA; ^3^ VRSA (MIC: 2–4 µg/mL)	Itching, pain and burning effects	Treatment of diabetic foot ulcers; topical	[23]
Mersacidin	Inhibition of cell wall	Gram (+), MRSA (MIC: 2–16 µg/mL); *Clostridium* spp. (MIC: 1–16 µg/mL)	Safety reported	Treatment of hospital-acquired infections	[54]
Planosporicin (Bacteriocin)	Inhibition of cell wall	Gram (+), (−); *Planomonospora* sp., MDR strains; *S. aureus* (MIC: 2–>128 μg/mL); *S. epidermidis*; *E. faecalis* (MIC: 32 μg/mL)	Not reported	Hospital-acquired infections	[55,56]
Plectasin (NZ2114)	Inhibition of cell wall synthesis	Gram (+), (−); MRSA (MIC: 16–32 μg/mL); *P. aeruginosa* (MIC >128 μg/mL)	Not reported	Pneumococcal peritonitis and pneumonia infections	[57,58]
**Clinical Phase**					
CZEN-002/(CKPV)2 (phase IIb)	Immunomodulation	*C. albicans*	Not reported	Vulvo-vaginal candidiasis; topical	[59]
D2A21 (phase III)	Membrane disruption	Gram (+), (−); fungi; Gram (+), (−); *S. aureus*; *E. coli*; *P. aeruginosa* (MIC: 4 µg/mL)	No side effects reported	Burn wound infections; topical	[60]
DPK-060 (phase II)	Membrane disruption and immunomodulation	Gram (+), (−); fungi; *S. aureus* (MIC: 4.6 µg/mL)	No side effects reported	Acute external otitis; ear drops	[23]
EA-230 (phase IIb)	Immunomodulation	Gram (−)	Safety reported	Sepsis and renal failure protection; IV	[61]
Histatin (phase I)	Membrane disruption	Gram (−); *C. albicans* (MIC: 4–16 μg/mL)	No side effects reported	Treatment of *P. aeruginosa* infections and oral candidiasis	[62,63]
hLF1-11 (phase I/II)	Membrane disruption	Gram(+), (−); fungi; *Staphylococcus* spp. (including MRSA), *Streptococcus mitis* (MIC: 1.6–6.3 μg/mL); *A. baumannii*, *Pseudomonas* spp., *Klebsiella* spp., *E. coli* (MIC: 6.3–12.5 μg/mL); *Candida* spp. (MIC >12.5 μg/mL)	Little discomfort at the injection site	LPS-related fungal infections; IV	[64,65]
IDR-1 (phase I)	Immunomodulation	MRSA; VRSA	No side effects reported	Infection prevention in immunocompromised patients	[62]
IMX942/SGX942 (IDR-1 derivative; phase III)	Immunomodulation	Gram (+), (−)	Safety reported	Treatment of nosocomial infections, neutropenia	[62]
LL-37 (phase IIb)	Barrel-stave mechanism of membrane disruption; inhibit ^5^ LPS binding	Bacteria, fungi and viral pathogens; *P. aeruginosa* (MIC: 64 μg/mL)	Cytotoxic	Diabetic foot ulcers; chronic middle ear infection	[25,66]
LTX-109 (Lytixar, phase II)	Membrane disruption and cell lysis	Gram (+); MRSA; VISA; VRSA (MIC: 2–4 µg/mL)	Itching, pain and burning effects	Treatment of nasal MRSA infections; nasal and topical	[67,68]
Mel4 (phase II/III)	Membrane disruption	Gram (+), (−); *P. aeruginosa* (MIC: 62.5–250 μg/mL)	No cytotoxicity and no resistance reported; no staining of human cornea	Contact lenses	[69,70]
Melimine (phase I/II)	Membrane disruption	Gram (+), (−); *P. aeruginosa* (MIC: 250–500 μg/mL)	No cytotoxicity and no resistance reported; staining of human cornea	Contact lenses	[69,70]
MX-226 (Omiganan^®^; phase III)	Cell disruption	Gram (+), (−); MRSA (MIC: 2–8 μg/mL); *E. coli* (MIC: 8–16 μg/mL); *C. albicans* (MIC: 64 μg/mL)	Not reported	Prevention of device-related infections; topical	[71]
Novexatin (NP213; phase IIb)	Membrane disruption	Fungi	Not reported	Treatment of dermatophyte fungal infections	[57]
Opebacan (rBPI21, neuprex; phase II)	Membrane disruption	Gram (+), (−)	Fever reported	Meningococcal, wound, and burn infections; IV	[15]
OP-145 (LL-37 derived; phase II)	Membrane disruption	Gram (+)	Lytic to human cells at high concentrations	Treatment of chronic bacterial middle ear infection; ear drops	[72]
PAC113 (P113; histatin 5 analog; phase IIb)	Membrane disruption and immunomodulation	Gram (+), (−); *Candida* spp.; *A. baumannii* (MIC: 38 μM); *P. aeruginosa* (MIC: 47 μM); *E. cloacae* (MIC: 90 μM)	No cytotoxicity reported	Oral candidiasis in HIV patients and prevention of bacterial periodontal disease; topical	[23,73]
p2TA (AB103; phase III)	Immunomodulation	Gram (−)	No adverse effects reported	Necrotizing soft tissue infections; IV	[74]
Ramoplanin (NTI-851; phase II)	Membrane disruption and cell wall synthesis	Gram (+); *C. difficile*; ^4^ MSSA (MIC: 2–4 μg/mL)	Low local tolerability when injected IV	Treatment of *C. difficile*-associated infections; oral	[75,76]
**FDA Approved**					
Anidulafungin (Eraxis™) in 2006	Inhibition of (1,3)-β-d-glucan synthase	Fungi; *C. albicans* and *K. crusei* (MIC: 0.06 μg/mL)	Hypersensitivity; hepatic effects	Treatment of *Candida* infections, especially esophageal candidiasis; IV infusion	[77,78]
Caspofungin (Cancidas) in 2001	Inhibition of β (1,3)-d-glucan production	Fungi; *C. albicans*; (MIC: 0.06 μg/mL); *K. crusei* (MIC: 0.25 μg/mL)	Hypersensitivity; hepatic effects	Treatment of esophageal candidiasis; IV	[79]
Dalbavancin (Dalvance™) in 2014	Inhibition of bacterial cell wall synthesis	Gram (+); *S. aureus* (MIC: ≤0.008–0.5 μg/mL)	May cause nausea, headache, and diarrhea	Treatment of complicated skin and skin structure infections (cSSSI); IV injection	[80,81]
Daptomycin (Cubicin^®^) in 2003	Membrane lytic	Gram (+); MRSA (MIC: 0.25–1 µg/mL); VISA (MIC: 1–8 µg/mL); VRSA (MIC: 0.12–1 µg/mL)	Not approved for pediatric patients	Treatment of cSSSI; IV injection	[68,82]
Gramicidin (Neosporin^®^) in 1955	Pore-forming; aggregation; membrane disruption	Gram (+), (−); *E. faecalis* (MIC: 8–16 μg/mL)	Hemolytic activity	Treatment of bacterial conjunctivitis; ointment	[44,83]
Polymyxins (Polymyxin E = colistin) in 1964	Membrane disruption	Gram (−); *P. aeruginosa* (MIC: 8 μg/mL); *E. coli* (0.5 μg/mL)	Used only as “last-resort” due to neuro- and nephrotoxic effects and neuromuscular blockage	Skin burns, for washing of wounds after operations, in superficial eye infections, and to protect minor wounds against infections; cream, ear and eye drops	[84,85]
Oritavancin (Orbactiv^®^) in 2014	Inhibition of bacterial cell wall synthesis and disruption of bacterial membrane ^1^	Gram (+); MRSA and MSSA (MIC: ≤0.008–0.25 μg/mL)	Long-term treatment is ambiguous	Treatment of (cSSSI); IV	[86,87]
Telavancin (Vibativ™ and Vibativ^®^) in 2009	Inhibition of bacterial cell wall synthesis and disruption of bacterial membrane ^1^	Gram (+); VRSA (MIC: 2–4 μg/mL); VISA (MIC: 0.25–1 μg/mL)	May induce acute kidney injury	Treatment of cSSSI; IV	[86,88]
Vancomycin (Vancocin^®^ HCl) in 2016	Inhibition of bacterial cell wall synthesis	Gram (+); *Candida* spp. (MIC: >256 µg/mL); MRSA (MIC: 0.5–2 µg/mL); VISA (MIC: 4–8 µg/mL); VRSA (MIC: 32–>64 µg/mL)	May cause nephrotoxicity	Treatment of severe MRSA infections; IV and oral	[68,82]

^1^ Oritavancin and telavancin may also act by membrane-pore, channel formation, or lysis of the cell membrane; ^2^ VISA: vancomycin-intermediate *S. aureus*; ^3^ VRSA: vancomycin-resistant *S. aureus*; ^4^ MSSA: methicillin-susceptible *Staphylococcus aureus*; ^5^ LPS: lipopolysaccharides; Gram (+): Gram-positive bacteria; Gram (−): Gram-negative bacteria. MIC: minimal inhibitory concentration.

**Table 3:** AMP’s advantages versus their disadvantages.

Advantages	Disadvantages
Several AMPs show broad and simultaneous activities against bacteria, fungi, viruses, and in case of infection with multiple microorganisms, one AMP could be efficient to overcome this issue.	AMPs are susceptible to proteolytic degradation, which leads to the loss of biological activity.
Natural AMPs are already found in high doses at the site of infection.	Some AMPs can be toxic to mammal cells at high concentrations.
Some AMPs have wound-healing and angiogenesis promotion properties, which are essential in case of hard-to-heal and infected wounds, such as diabetes and foot ulcer.	AMPs can induce hypersensitivity reactions after application.
AMPs show as well anti-inflammatory properties by modulating immune cytokines, which are responsible for the inflammatory response.	AMPs can be influenced by pH variation and at low concentration of salt can be destabilized, leading to loss of activity.
AMPs exhibit therapeutic antimicrobial activity at extremely low concentrations in the microscale and sometimes nanoscale range.	AMP’s production and purification costs are high.
Resistance to AMPs is very low and only in a limited number of AMPs.	
AMPs showed to be time-efficient; some AMPs can act within a few seconds to a few minutes.	
AMPs inhibit biofilm formation, which is especially important to prevent bacterial growth on medical devices.	
AMPs show synergism when chemically coupled to polymers, encapsulated into different delivery systems or simultaneously applied with antimicrobial agents.	
AMPs can act synergistically with antibiotics.	
